# Astrocyte dysfunction and neuronal network hyperactivity in a CRISPR engineered pluripotent stem cell model of frontotemporal dementia

**DOI:** 10.1093/braincomms/fcad158

**Published:** 2023-05-18

**Authors:** Isaac Canals, Andrea Comella-Bolla, Efrain Cepeda-Prado, Natalia Avaliani, James A Crowe, Leal Oburoglu, Andreas Bruzelius, Naomi King, María A Pajares, Dolores Pérez-Sala, Andreas Heuer, Daniella Rylander Ottosson, Jordi Soriano, Henrik Ahlenius

**Affiliations:** Stem Cells, Aging and Neurodegeneration group, Department of Clinical Sciences, Neurology, Faculty of Medicine, Lund University, 22184, Lund, Sweden; Lund Stem Cell Center, 22184, Lund, Sweden; Glial and Neuronal Biology lab, Department of Experimental Medical Science, Faculty of Medicine, Lund University, 22184, Lund, Sweden; Stem Cells, Aging and Neurodegeneration group, Department of Clinical Sciences, Neurology, Faculty of Medicine, Lund University, 22184, Lund, Sweden; Lund Stem Cell Center, 22184, Lund, Sweden; Lund Stem Cell Center, 22184, Lund, Sweden; Regenerative Neurophysiology group, Department of Experimental Medical Science, Faculty of Medicine, Lund University, 22184, Lund, Sweden; Lund Stem Cell Center, 22184, Lund, Sweden; Lund Stem Cell Center, 22184, Lund, Sweden; Glial and Neuronal Biology lab, Department of Experimental Medical Science, Faculty of Medicine, Lund University, 22184, Lund, Sweden; Lund Stem Cell Center, 22184, Lund, Sweden; Hematopoietic Stem Cell Development group, Department of Laboratory Medicine, Faculty of Medicine, Lund University, 22184, Lund, Sweden; Lund Stem Cell Center, 22184, Lund, Sweden; Regenerative Neurophysiology group, Department of Experimental Medical Science, Faculty of Medicine, Lund University, 22184, Lund, Sweden; Behavioural Neuroscience Laboratory, Department of Experimental Medical Science, Faculty of Medicine, Lund University, 22184, Lund, Sweden; Department of Structural and Chemical Biology, Centro de Investigaciones Biológicas Margarita Salas, C.S.I.C., 28040, Madrid, Spain; Department of Structural and Chemical Biology, Centro de Investigaciones Biológicas Margarita Salas, C.S.I.C., 28040, Madrid, Spain; Behavioural Neuroscience Laboratory, Department of Experimental Medical Science, Faculty of Medicine, Lund University, 22184, Lund, Sweden; Lund Stem Cell Center, 22184, Lund, Sweden; Regenerative Neurophysiology group, Department of Experimental Medical Science, Faculty of Medicine, Lund University, 22184, Lund, Sweden; The Neurophysics group, Departament de Física de la Matèria Condensada, Universitat de Barcelona, 08028, Barcelona, Spain; Universitat de Barcelona Institute of Complex Systems (UBICS), 08028, Barcelona, Spain; Stem Cells, Aging and Neurodegeneration group, Department of Clinical Sciences, Neurology, Faculty of Medicine, Lund University, 22184, Lund, Sweden; Lund Stem Cell Center, 22184, Lund, Sweden

**Keywords:** human pluripotent stem cells, CRISPR/Cas9 genome editing, frontotemporal dementia, electrophysiology, neuronal network analysis

## Abstract

Frontotemporal dementia (FTD) is the second most prevalent type of early-onset dementia and up to 40% of cases are familial forms. One of the genes mutated in patients is *CHMP2B*, which encodes a protein found in a complex important for maturation of late endosomes, an essential process for recycling membrane proteins through the endolysosomal system. Here, we have generated a *CHMP2B*-mutated human embryonic stem cell line using genome editing with the purpose to create a human *in vitro* FTD disease model. To date, most studies have focused on neuronal alterations; however, we present a new co-culture system in which neurons and astrocytes are independently generated from human embryonic stem cells and combined in co-cultures. With this approach, we have identified alterations in the endolysosomal system of FTD astrocytes, a higher capacity of astrocytes to uptake and respond to glutamate, and a neuronal network hyperactivity as well as excessive synchronization. Overall, our data indicates that astrocyte alterations precede neuronal impairments and could potentially trigger neuronal network changes, indicating the important and specific role of astrocytes in disease development.

## Teaser

Canals *et al*. report a new disease model of Frontotemporal Dementia generated with genome editing of the *CHMP2B* gene in human embryonic stem cells, differentiated to induced neurons and astrocytes, and generating neuron-astrocyte co-cultures to identify impaired astrocytic glutamate homeostasis and evident alterations in neuronal network connectivity and synchronization.

## Graphical Abstract

**Graphical abstract:**
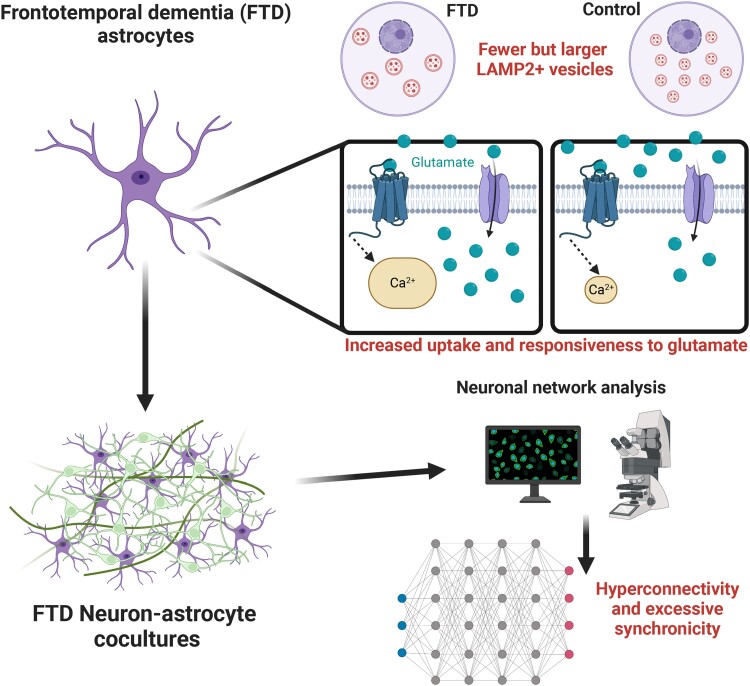

## Introduction

Frontotemporal dementia (FTD) is one of the most common types of early-onset dementia with an estimated prevalence of 3% to 26% in patients under 65 years old, and it is characterized by a profound degeneration of the frontal and temporal lobes of the brain.^1^ Around 40% of the cases are genetic, caused by mutations in genes such as *C9ORF72*, *MAPT*, *GRN*, or less frequently *CHMP2B.*^2^ Interestingly, most genes mutated in FTD patients are related to the endolysosomal and autophagy pathways, responsible for clearing aggregates and dysfunctional organelles, highlighting the fragility of neurons to alterations in these systems.

*CHMP2B* is a gene located in the short arm of chromosome 3, contains six exons and encodes a protein that is a component of the endosomal sorting complex required for transport-III (ESCRT-III), essential for maturation of early endosomes towards multivesicular bodies.^3^ The first time that a dominant mutation in *CHMP2B* was identified as a cause for FTD was in a Danish family,^4^ where a change in the acceptor splicing site of intron 5 led to aberrant splicing of the mRNA, causing enlarged endosomes^5^ and affecting the fusion of endosomes with lysosomes.^6^

To date, most *in vitro CHMP2B-*FTD models have been generated using cultured mouse hippocampal neurons transfected either with constructs expressing different isoforms generated by the intron 5 mutation or short hairpin RNAs to inhibit endogenous expression of *Chmp2b*. In these models, overexpression of mutant *CHMP2B* promotes formation of aggregates and affects density, morphology, and maturation of synaptic spines, with *Chmp2b* depletion displaying similar effects and confirming its essential role for synapses.^7,8^ In a transgenic mouse model overexpressing the human intron 5 mutation, progressive inclusions, axonal swellings containing endolysosomal vesicles^9^, and accumulation of autofluorescent aggregates within endolysosomes,^10^ together with loss of brain volume, neuroinflammation, and social and motor deficits^11^ have been identified, highlighting the progressive character of FTD. In a later study, using primary neuronal cultures derived from this animal model, impaired endolysosomal trafficking was found,^12^ leading to decreased expression of miR-124, which regulates the composition of alpha-amino-3-hydroxy-5-methyl-4-isoxazole propionic acid (AMPA) receptors subunits in the forebrain.^13^

Although important to investigate disease mechanisms of *CHMP2B*-FTD, these studies were performed in murine models and using overexpression or depletion of CHMP2B protein levels, which do not resemble pathophysiology in patients. In addition, lower degree of complexity of murine brains and neural cells compared to human counterparts, as well as different physiology, could lead to disparity of specific phenotypes and disease mechanisms. The establishment of induced pluripotent stem cell (iPSC) technology represented a unique opportunity to facilitate studies of human brain disorders. In the case of *CHMP2B*-FTD, iPSC lines were recently generated from fibroblasts of three FTD patients, and isogenic controls were obtained using clustered regularly interspaced short palindromic repeats (CRISPR)/CRISPR associated protein 9 (Cas9) genome editing to correct the causative mutation.^14^ These iPSCs were differentiated into forebrain cortical neurons that presented enlarged endosomes and abnormal mitochondria leading to increased oxidative stress together with an imbalance of iron homeostasis. In a subsequent study, authors identified dysregulation of the glutamate–glutamine pathways in neurons and increased glutamate uptake in astrocytes.^15^

All these studies provided valuable information on molecular impairments occurring in brain cells, however, there is a lack of studies on alterations affecting neuronal functionality and neuronal networks in human models. Brain imaging scanning has shown that atrophy can appear even before clinical onset of the disease,^16^ and a recent study on FTD patients using transcranial magnetic stimulation, showed circuit alterations in presymptomatic patients 15 years before the expected onset of the disease.^17^ For these reasons and to complement newly generated iPSC models of *CHMP2B*-FTD, in this work, we generated a human embryonic stem cell (hESC)-based co-culture model of excitatory neurons and astrocytes to investigate functional alterations at the cellular and network level in FTD caused by *CHMP2B* mutations. We present a system that allowed to analyze neuron-astrocyte co-cultures at a time point in which endolysosomal alterations appear and revealed altered response and regulation of extracellular glutamate by astrocytes, and dysregulated neuronal network connectivity and synchronicity.

## Materials and methods

### CRISPR/Cas9 genome editing

CRISPR/Cas9 genome editing was performed as previously described.^18^ Briefly, hESCs were dissociated with Accutase, and 300 000 cells were plated in a matrigel coated well of a 6-well plate with mTeSR1 medium supplemented with 10 µM ROCK inhibitor one day before transfection. The day after, medium was changed and fresh mTeSR1 medium supplemented with 10 µM ROCK inhibitor was added. Cells were transfected using 8 µl of FuGene (Active Motif), with 1 μg of the px459 Cas9 vector (Addgene #62988) carrying a single-guide RNA (sgRNA) targeting the CHMP2B sequence near the site of the intron 5 mutation (Supplementary Table 1) and 0.5 µg of each 75-bp donor single-stranded oligodeoxynucleotide (ssODN) carrying either the intron 5 mutation and a G > A change in the protospacer adjacent motif (PAM) sequence of the sgRNA or the wild type (WT) sequence together with the change in the PAM (Supplementary Table 1). Both ssODNs had two phosphorothioate bonds between the last three nucleotides at each end to protect the donor strands from digestion by exonucleases. The day after transfection, a 48-h selection with 1.25 ng/ml of puromycin started and 10 µM of ROCK inhibitor was added to improve survival. Surviving cells were dissociated after 7–14 days, and 50 or 100 cells were plated with mTeSR1 medium containing 10 μM ROCK inhibitor in six-well plates coated with laminin-521 (BioLamina). When evident, colonies were picked and plated in a 24-well plate with mTesR1 medium containing 10 μM ROCK inhibitor and expanded. Clones were analysed after DNA extraction with a DNeasy Blood and Tissue Kit (Qiagen). Using specific primers (Supplementary Table 2), a 269-bp region of the *CHMP2B* gene was amplified to assess incorporation of the mutation by sequencing. For off-target effects, predicted genomic locations of interest were amplified with specific primers (Supplementary Table 2). Sequence data were analysed using SnapGene (GSL Biotech).

### Neuronal and astrocyte differentiation

Generation of lentivirus and differentiation of the Control and FTD-hESC lines towards induced neurons (iNs) and induced astrocytes (iAs) were performed as previously described.^19,20^ Briefly, 250.000 (iN) or 350.000 (iAs) hESCs were plated on Matrigel-coated 6-well plates and infected with 1 µl of each virus (rtTA and Ngn2 for iNs and rtTA, Sox9 and Nfib for iAs) in mTeSR1 supplemented with 8 µg/ml of Polybrene. Twenty-four hours later, medium was changed and fresh mTeSR1 supplemented with 2.5 µg/ml doxycycline was added. Subsequently, selection was conducted for 3 (Puromycin) or 5 (Hygromycin) days in specific medium for iNs or iAs. At day 7 after induction, co-cultures were prepared on 13-mm coverslips as previously described^21^ with a total of 100.000 or 150.000 cells (74% iNs and 26% iAs), on 6-well plates coated with Matrigel with a total of 10^6^ cells (74% iNs and 26% iAs) or alternatively on 6-well plates with inserts (413.000 iNs on insert and 332.000 iAs in the well) coated with Matrigel. Plasmids used are listed in Supplementary Table 3.

### Electrochemical detection of glutamate

Co-cultures at 49 days of differentiation on coverslips were transduced with pLenti-CaMKIIa-ChR2-eYFP using 1 µl of virus in 500 µl of medium one week before recordings. Ceramic microelectrode arrays (MEAs) with four Pt recording sites (S2 configuration; CenMet, KY, USA) were prepared as described previously.^22,23^ In brief, two adjacent recording sites were coated with L-glutamate oxidase (GluOx), bovine serum albumin (BSA), and glutaraldehyde. GluOx is an enzyme that breaks down glutamate to α-ketoglutarate and H_2_O_2_. The remaining two recording sentinel sites were coated with protein matrix (BSA and glutaraldehyde) only. This configuration allows detection of glutamate from GluOx coated sites whereas sentinel sites only detect background current. Amperometric recordings were conducted using a potential of +0.7 V versus an Ag/AgCl reference electrode on the Pt surface which results in a two-electron oxidation of H_2_O_2_. To prevent other electroactive molecules from reaching the recording surface a size-exclusion layer of 1,3-Phenylendiamine was electroplated on the day of recording. The generated current was amplified using a Headstage (500×) and digitized with the F.A.S.T. 16mkIII recording equipment (Quanteon, KY, USA). After calibration, only electrodes with a limit of detection <1 µM, a linearity of *R*^2^ > 0.95, and a selectivity > 100:1 were used for recordings. For data analysis, the current recorded from the sentinel sites (background current) was subtracted from the GluOx coated sites (glutamate current + background current) to obtain the self-referenced glutamate recording trace. For stimulation of optogenetic receptors, a 50 µM optical fibre cable (Thorlabs) was mounted to the recording electrode^24^ and connected to a 473 nM blue DPSS laser calibrated before each recording. Cell culture plates were quickly transferred from the incubator onto a water heated pad (Micro Temp, model 749) set to 37°C. An Ag/AgCl reference electrode and the enzyme-coated MEA were inserted into the medium and left for 60 min to stabilize. For each recording, we stimulated glutamate release three times for 60 s with 5-min intervals using 1.25 mW laser intensity at constant stimulation.

### Electrophysiology analysis

Whole-cell patch-clamp recordings were obtained at days 40–50 from co-cultures of iN and iAs, or iAs cultured alone. Coverslips with cultures were transferred to a recording chamber continually perfused at 1 ml/min with artificial cerebrospinal fluid (aCSF) containing (in mM): 119 NaCl, 2.5 KCl, 1.3 MgSO_4_, 2.5 CaCl_2_, 25 Glucose and 26 NaHCO_3_, and gassed with 95% O_2_–5% CO_2_ (carbogen gas) at a RT (pH ∼7.4, osm 305 ∼mOsm). The cells were visualised using a fixed stage Olympus Microscope (BX51WI) coupled with an IR-CCD camera and a 40× water immersion objective. Patch pipettes (5–7 MΩ resistance) were pulled from standard borosilicate glass (BF150-86-10, O.D.: 1.5 mm, I.D.: 0.86 mm, with filament, Sutter Instrument, Novato, CA, USA) by using a micropipette puller (p1000, Sutter Instrument, Novato, CA, USA). Recording pipettes were filled with intracellular solution containing (in mM): K-gluconate 122.5, KCl 12.5, NaCl 8, HEPES 10, MgATP 2, and Na_3_GTP 0.3, pH adjusted with KOH to 7.4 and 290–295 mOsm. Whole-cell patch-clamp recordings were obtained using a Multiclamp 700B amplifier and pClamp 10.4 data acquisition software (Axon Instruments, Molecular Devices, USA). To minimize recording errors, series resistance was compensated to 60–80% and recordings with leaky patch-clamp seal currents >100 pA were excluded. This was performed during the initial test pulse immediately after opening the cell, at the holding potential of -80 mV and was monitored throughout the recording to make sure that it was not increasing significantly. Moreover, quality of recording was monitored with a test pulse throughout the recording and cells were discarded if the access resistance was over 30 MΩ. Resting membrane potential (RMP) was measured at the beginning of the experiments, right after breaking into the cell. The capacitance of the cell was directly obtained from pClamp 10.4 data acquisition software. The input resistance (Ri) was calculated from a 10 mV pulse and monitored throughout the experiment.

For iN recordings, the action potential (AP) threshold, peak amplitude, after hyperpolarisation and AP halfwidth were measured from the first observed spike evoked by the rheobase current injection step. Membrane currents were elicited in the voltage-clamp mode at the holding potential of -70 mV. Sodium and potassium currents were evoked by stepwise injection of depolarizing potentials from -70 mV to +40 mV (10 mV increments). Spontaneous synaptic activity was recorded in voltage-clamp mode at -70 mV and for recording miniature excitatory postsynaptic currents (mEPSCs), sodium channels were selectively blocked using tetrodotoxin (TTX 1 M, Tocris, Bristol, UK). N-methyl-D-aspartate receptor and AMPA/kainate receptor antagonists, D-2-amino-5-phosphonovalerate (D-APV, 50 µM, Tocris, Bristol, UK) and 2,3-dioxo-6-nitro-1,2,3,4-tetrahydrobenzo[*f*]quinoxaline-7-sulfonamide (NBQX, 50 µM, Tocris, Bristol, UK), respectively, were added to the extracellular solution in some recordings for blocking the excitatory synaptic currents. For iAs, in addition to passive membrane properties, potassium currents were elicited by de- and hyperpolarisation steps between -170 and +50 mV (10 mV increments) at the holding potential of -80 mV.

Data were analysed offline using Clampfit 10.3 (Axon Instruments, Molecular Devices, USA), Mini Analysis Program (Synaptosoft) and Igor Pro 8.04 (Wavemetrics, Portland, OA, USA).

### Calcium imaging

Calcium imaging recordings for iNs were carried out at 49 days *in vitro* to assess spontaneous activity of iNs in co-cultures with iAs. Recordings were performed in aCSF. The fluorescent Ca^2+^ indicator Fluo4-Acetoxymethyl ester (494/506 nm) (F14201; Thermo Fisher Scientific Inc.) was used in all measurements. One millimolar Fluo4 was dissolved in dimethyl sulfoxide (DMSO) and Pluronic F-127 (20% solution in DMSO) (1:1), added to aCSF to a final concentration of 2 μM and incubated in the dark for 30 min at 37°C. After incubation, cultures on coverslips were placed in the recording chamber mounted on an Olympus Wide field epifluorescence microscope equipped with an Orca Flash 2.8 CMOS camera (Hamamatsu Photonics), a light-emitting diode light source for fluorescence, and a heating and perfusion system to exchange the recording solution continuously. Image sequences were acquired using the HCImage LIVE software with a speed of 20 frames per second for 10 min, with a size of 1920 × 1440 pixels, and microscope settings combined a 10 × objective with a 0.63 × optical zoom, providing a spatial resolution of 4.40 μm/pixel and a field of view (FOV) of 4.2 × 3.2 mm^2^ that contained >200 cells. Recordings were carried out at 34°C.

Calcium imaging recordings for iAs were carried out at 42 days *in vitro* to assess response to glutamate. Cells were plated in 24-well plates and recordings were performed in Phenol-red free Neurobasal. Fluo4 was added to Neurobasal for a final concentration of 2 μM and incubated in the dark for 30 min at 37°C. After incubation, cells were washed once with fresh Neurobasal and imaged immediately. Live fluorescence imaging was carried out in a Zeiss LSM 780 confocal microscope with an incubation chamber for 37°C and 5% CO_2_. Images were taken every 1 s with a 10 × objective for 2 min before addition of 200 µM glutamate, and for 3 min after stimulation.

### Calcium imaging data analysis

Fluorescence recordings were analyzed with the custom-made software NETCAL.^25^ For each experiment, about 150–300 regions of interest (ROIs) were automatically selected on the FOV of the culture based on their brightness and roundness and ascribed as iNs. The fluorescence level for each ROI was then extracted as a function of time, providing the set of fluorescence traces of the cells in culture. Traces were next smoothed out to reduce noise and corrected for small drifts through a moving window filter in combination with a high-band filter.^26^ The drift correction effectively removed large time-scale fluctuations while preserving sharp peaks in fluorescence that revealed neuronal activity. The fluorescence trace *F*_i_(*t*) for each cell *i* was then normalized as %DFF_i_ = 100·(*F*_i_(*t*)-*F*_i,0_)/*F*_i,0_, where *F*_i,0_ is the fluorescence trace of the cell at rest.

For iAs, we analyzed the response of astrocytes to glutamate by extracting the fluorescence trace for those astrocytes that responded immediately (within 1 s) to glutamate, and then averaged out the collection of traces (99 for control and 125 for FTD).

### Calcium imaging trace classification

iNs were separated from iAs based on their fluorescence profiles by means of a supervised machine learning algorithm.^27^ First, a subset of traces was manually selected for each group (iNs and iAs). Then, an ensemble classifier was trained with this subset using adaptive boosting.^27^ The classifier uses a set of features obtained from the statistical moments of the traces (average, variance, or skewness, see^25^ for details).

### Spike train inference and neuronal properties

A sharp increase in the fluorescence signal revealed activity. The train of spike onset times for each iN was extracted using the Oasis method^28^ and based on deconvolution of the fluorescence signal. For single-spike detection, threshold method and Ar1 model were used along with a lambda of 5 and a minimum spike signal of *S*_min_ = 0.3. The spiking events for all iNs were represented in the form of raster plots and used to carry out all statistical analyses. A recording typically contained about 150–300 iNs. Neuronal properties were assessed by extracting a set of spike and burst features. The latter was defined as concatenated spikes with intervals smaller than 1 s. Firing rate (spikes along time, Hz); inter-spike interval (ISI, s); bursting rate (bursts along time, Hz); number of spikes within a burst; ISI inside a burst; inter-burst interval (IBI, s); bursts duration (s); and bursts amplitude were the activity descriptors analyzed.

### Global network activity (GNA), network bursts, and distribution of burst amplitudes

GNA quantified the degree of coordinated activity in a neuronal network, i.e. the capacity of a neuronal ensemble (from few neurons to the entire culture) to activate together in a short time window. GNA was computed as the number of neurons in the network that activated together in a sliding window 2 s wide and 0.5 s step, diving afterwards by the number of active neurons in the network. Thus, GNA varied between 0 (no activity) and 1 (full network activation). Peaks in the GNA profile identified network bursts, i.e. neuronal coordinated activations that encompassed a substantial part of the network. A network burst was deemed significant whenever its amplitude *A*_b_ was above the mean + 3 standard of background activity. All significant burst amplitudes *A*_b_, across replicates and for a given experimental condition, were pooled together to build the distribution of amplitudes. These distributions were finally compared between Control and FTD to determine the tendency of networks towards strong bursting.

### Functional connectivity

Causal relationships among pairs of neuronal spike trains were computed using a modified version of Generalized Transfer Entropy (GTE)^29,30^ run in MATLAB. Binarized spike trains (‘1’ for the presence of a spike, ‘0’ for absence) were constructed for each neuron, and filtered to remove concatenated activations and that could generate artifacts in the inferred functional networks .^29^ An effective connection was then established between neurons X and Y whenever the information contained in X significantly increased the capacity to predict future states of Y. Instant feedback was present, and Markov Order was set as 2.^30^ A connection was deemed significant whenever the GTE estimate exceeded the mean + 2 standard deviations of the joint distribution, i.e. the set of GTE scores of all inputs to Y and all outputs to X. The GTE scores were finally set to 0 (absence of connection) or 1 (connection present), shaping binary and directed connectivity matrices. For clarity, the term ‘functional’ was used instead of ‘effective’ throughout the description of results.

### Network measures

The obtained functional connectivity matrices were analyzed using the ‘Brain Connectivity Toolbox’,^31^ in MATLAB, to quantify their topological organization. The most important extracted network measures were: (i) average connectivity, obtained as the mean number of functional connections per neuron, and excluding non-connected neurons; (ii) distance between connected neurons, which corresponds to the average Euclidean distance between functionally connected neurons; (iii) the Global Efficiency *G*_EFF,_^32^ which accounts for the capacity of neurons to exchange information across the entire network (*G*_EFF_≅0 for low global communication and *G*_EFF_≅1 for very strong); (iv) the Local Efficiency *L*_EFF,_^31^ which reflects the capacity of the neurons to communicate within their neighborhood (*L*_EFF_≅0 for low local communication and *L*_EFF_≅1 for very strong); (v) and the Modularity *Q*,^33^ which accounts for the tendency of neurons to form functional modules, i.e. groups of neurons more connected within their groups than with the rest of the network (*Q*≅0 indicates than the whole network shapes a single module, while *Q*≅0.3 or larger indicates a clear presence of modules).

### Statistical analysis

Data for all assays is presented as mean ± standard error of the mean (SEM) of 3–4 independent experiments. Statistical tests were performed using GraphPad Prism software, comparing the Control sample to FTD. Shapiro–Wilk normality test was carried out before assuming normal distribution of the data. Statistical analyses used for each experiment are stated in the corresponding figure legends. For all tests, significance was set at *P* < 0.05.

### Data availability

All information necessary to evaluate findings is included in the paper. Additional data can be provided upon request. The FTD-hESC lines generated here will be made available upon reasonable request.

## Results

### Generation of a genome engineered FTD-hESC line carrying the intron 5 mutation

To investigate cell-specific alterations in FTD triggered by mutations in the *CHMP2B* gene, we generated a hESC line carrying the previously described intron 5 mutation^4^ in heterozygosis, as it is found in patients for being a dominant mutation, using CRISPR/Cas9 genome editing. First, we identified *in silico* four different sgRNA targets near the genomic region of the intron 5 mutation and assessed their efficiency in hESCs, selecting sgRNA4 (Supplementary Table 1) as the most efficient. Next, we transfected hESCs with a vector containing sgRNA4 and Cas9 together with an ssODN to promote homology-directed repair (HDR) and to introduce the intron 5 mutation (Supplementary Fig. 1A). To improve efficiency of HDR, the ssODN was designed with short homology arms, complementary to the sgRNA sequence,^34^ with phosphorothioate modifications in the last two nucleotide bonds at each side to protect from degradation by exonucleases,^35^ and including a synonymous change in the PAM sequence to avoid repeated targeting. With this strategy, we were able to identify several edited clones, however, no heterozygous clones were found.

**Figure 1:**
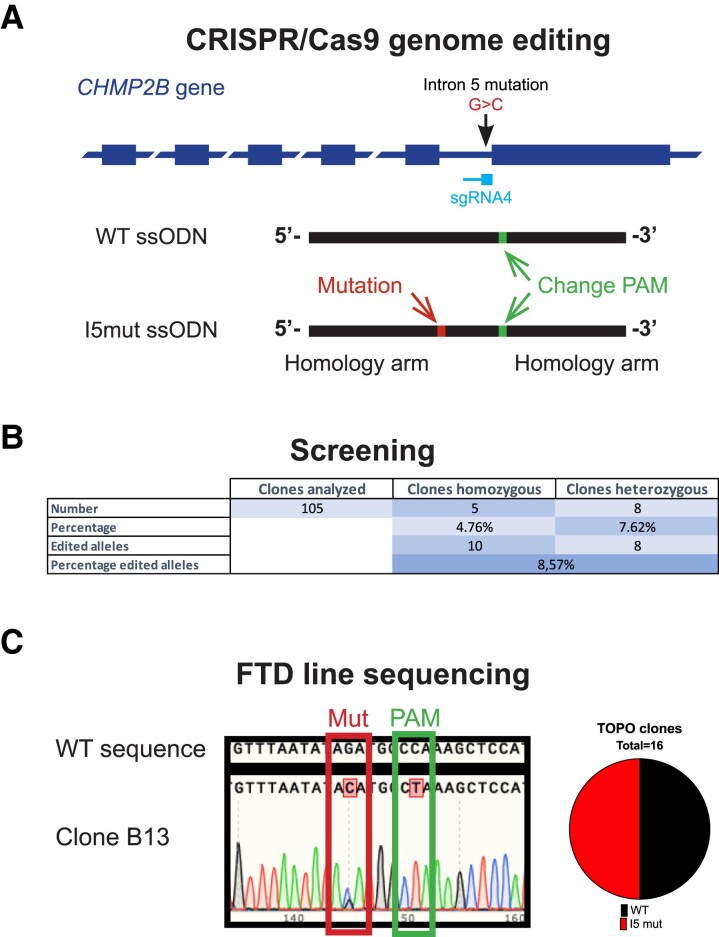
**Generation of CRISPR/Cas9 edited hESCs carrying the intron 5 mutation in *CHMP2B*. (A)** Schematic representation of the CRISPR/Cas9 strategy used to target and modify the *CHMP2B* gene. **(B)** Summary of results from screening clonal lines upon genome editing. **(C)** Sanger sequencing chromatogram of clone B13 (left panel) and results of TOPO clones after analysis of 16 samples.

The consistent presence of indels suggested very high efficiency of editing for sgRNA4, which did not leave any allele unedited. To solve this issue, we included a second ssODN harboring the normal WT allele but containing the change in the PAM sequence in our transfections (Fig. 1A). With this new strategy, we identified several clones carrying the mutation in homozygosis or heterozygosis (Fig. 1B) and, interestingly, the PAM sequence change in homozygosity, indicating that each ssODN had been used in one allele. We selected one clone for further characterization, hereafter referred to as FTD-hESC, in contrast to the parental line referred to as Control-hESC.

We confirmed heterozygosity of *CHMP2B* in the FTD-hESC line by Sanger sequencing, which revealed a double peak in the histograms at the mutation site (Fig. 1C). Furthermore, TOPO cloning of the PCR product and analysis of 16 bacterial clones showed that, as expected, 8 had the mutation and 8 the WT allele, while all had the change in the PAM sequence (Fig. 1C), confirming the heterozygosity of the line for the intron 5 mutation. Next, we carried out several assays to ensure quality of the FTD-hESC line and found a normal karyotype (Supplementary Fig. 1B), normal expression levels of pluripotency markers *NANOG* and *OCT3/4* at mRNA (Supplementary Fig. 1C) and protein levels (Supplementary Fig. 1D), and normal levels of proliferation and cell death (Supplementary Fig. 1E). Moreover, sequencing of *in silico* predicted top 5 candidate off-target sites for sgRNA4 (Supplementary Fig. 1F) showed no unexpected edits in those regions. Finally, we analyzed mRNA levels of *CHMP2B* with real-time quantitative PCR and found no differences between Control- and FTD-hESCs (Supplementary Fig. 2A), suggesting that the editing process or presence of the intron 5 mutation did not alter *CHMP2B* expression levels. To further demonstrate the presence and effect of the mutation, we performed PCR on cDNA generated from RNA extracted from control and FTD hESCs. This revealed a band corresponding to the inclusion of intron 5 in the mRNA only in the FTD line (Supplementary Fig. 2B). Interestingly, we could not detect any band that would correspond to the Δ10 alternative splicing, which has been previously shown to be present in very low amount in patient brains.^6^

Altogether, our results show that we successfully introduced an FTD causative mutation in the intron 5 of the *CHMP2B* gene of a hESC line in heterozygosity, as in patients, and without introducing any unwanted alteration in the genome or in essential properties of the pluripotent stem cells.

### Establishment of a FTD neuron and astrocyte co-culture system with astrocytic endolysosomal alterations

**Figure 2:**
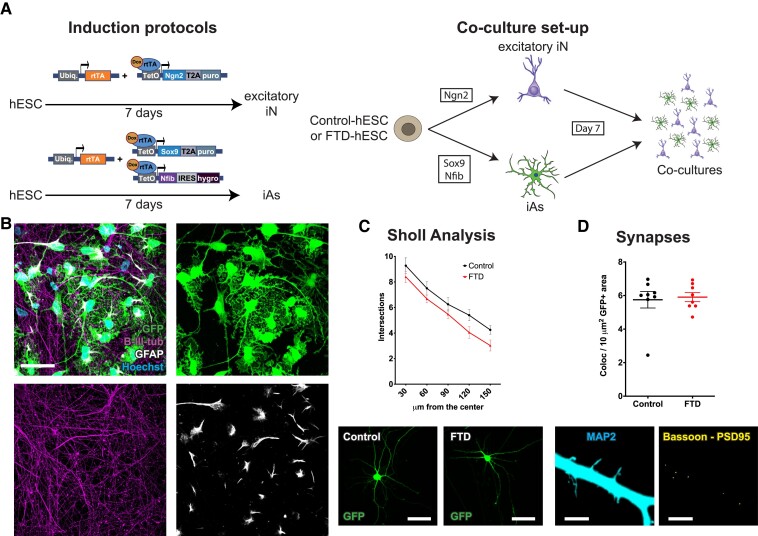
**Establishment of a co-culture system with neurons and astrocytes, and morphological and synaptic characterization of neurons. (A)** Schematic representation of protocols to generate induced neurons (iNs), induced astrocytes (iAs) and the co-culture system. Ubiq = ubiquitin, rtTA = reverse tetracycline transcriptional activator, Dox = doxycycline, tetO = tetracycline operator, Ngn2 = neurogenin 2, T2A = thosea asigna virus 2A sequence, puro = puromycin, Sox9 = SRY-Box transcription factor 9, Nfib = nuclear factor I B, IRES = internal ribosome entry site, hygro = hygromycin. **(B)** Representative image of an immunostained co-culture showing the distribution and complexity of neurons (beta-III-tubulin positive cells in magenta) and GFP-labelled astrocytes (GFP in green and GFAP positive cells in white). **(C)** Sholl analysis showing number of intersections at different distances from the soma (upper graph) and representative images of GFP-labelled Control and FTD neurons used for analysis (lower images). Data are presented as the mean ± SEM from eight independent experiments. A two-way ANOVA with Tukey’s multiple comparisons test was used to analyze significance (*F*_(4,70)_) = 0.1675; *P* = 0.9542). **(D)** Number of pre- and postsynaptic puncta colocalization per area of neuronal projection (upper graph) and representative images of threshold selection of a neurite (left image) and co-localizing areas in yellow of the presynaptic marker Bassoon and postsynaptic marker PSD95 (right image). Data are presented as mean ± SEM from eight independent cultures from four differentiations. A two-tailed Mann–Whitney test was used to analyze significance (*U* = 28; *P* = 0.7209). Scale bar = 50 µm (**B**), 100 µm (**C**), and 5 µm (**D**).

To facilitate the study of cell autonomous and non-autonomous phenotypes in FTD neurons and astrocytes, we sought to generate a co-culture system combining independently generated excitatory neurons and astrocytes (Fig. 2A). To differentiate hESCs to neurons, we used a previously developed protocol based on overexpression of the neurogenic transcription factor Ngn2, which generates a pure population of excitatory iNs.^20^ For differentiation to astrocytes, we overexpressed two gliogenic transcription factors, Sox9 and Nfib, following a protocol we recently developed, which allows for rapid generation of a pure population of iAs.^19^ We first analyzed upregulation of *MAP2* and *S100B* for neurons and astrocytes, respectively, at 7 days of differentiation and did not find any difference (Supplementary Fig. 2C), confirming that differentiation was not affected in FTD-hESCs. To confirm the forebrain fate of the iNs and astrocytes, we analyzed expression levels of markers for forebrain (FOXG1), midbrain (LMX1A), and hindbrain (HOXB4), showing that both iNs and iAs had higher expression levels of the forebrain compared to midbrain and hindbrain markers (Supplementary Fig. 2D). In addition, the intron 5 mutation did not affect *CHMP2B* mRNA levels in FTD-iNs or FTD-iAs compared to Control cells (Supplementary Fig. 2E).

We next generated co-cultures of iNs and iAs by combining them in a 3:1 ratio (Fig. 2A). At day 42 after induction (35 days after co-culture), iNs were positive for beta-III-tubulin while iAs were positive for glial fibrillary acidic protein, specific markers for each cell type. In addition, labeling of iAs with green fluorescent protein (GFP) showed complex stellate morphology (Fig. 2B), as originally reported using this protocol.^19^ Previous studies in cultured mouse neurons have shown that overexpression of the intron 5 mutation increases while *Chmp2b* knock-down reduces dendritic arborization.^7,12^ To investigate how the intron 5 mutation affects morphology in human neurons, we used sparse GFP-labelling. Both Control- and FTD-iNs showed complex morphologies with several branches and using Sholl analysis we detected a slight decrease in number of branches in FTD-iNs, although these differences were not significant (Fig. 2C). In addition, and considering synaptic alterations described in cultured mouse neurons,^8^ we carried out co-localization analysis of Bassoon (presynaptic marker) and PSD-95 (postsynaptic marker) to quantify number of synapses, however, we did not detect any difference between Control- and FTD-iNs (Fig. 2D).

**Figure 3:**
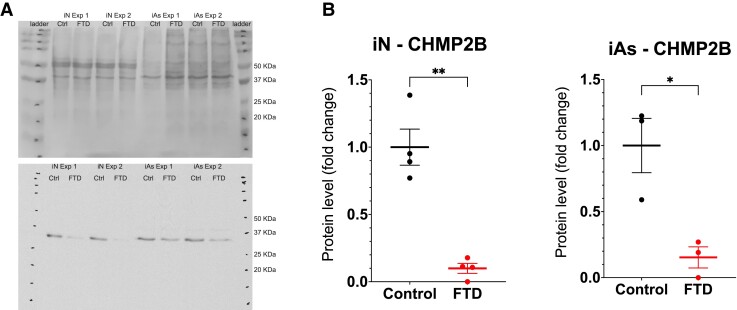
**CHMP2B is present in low amounts at the protein level in FTD-iN and FTD-iAs.** (**A**) Representative Western blots including iN and iAs from two different experiments (Exp) with Ponceau staining for normalization (upper gel) and CHMP2B staining (lower gel). (**B**) Quantification of the fold change in CHMP2B levels normalized towards Ponceau staining for iNs and iAs derived from Control and FTD lines. Data are presented as mean ± SEM from three to four independent experiments. A *t*-test with Welch’s correction was used to analyze significance. *P ≤ 0.05, **P ≤ 0.01.

Due to the discrepancy between our results and previous data from animal models for the synaptic alterations, we decided to examine the levels of CHMP2B protein in both iN and iAs after 42 days of differentiation using Western blot (Fig. 3A). To be able to independently analyze each cell type, cells were grown using inserts, which allow for co-cultures but without physical neuron-to-astrocyte contact. Our results showed that CHMP2B levels are drastically reduced, in both neurons and astrocytes derived from the FTD line, below what is expected from normal protein levels from one allele (Fig. 3B). Interestingly, we could not detect any of the truncated isoforms of the protein.

**Figure 4:**
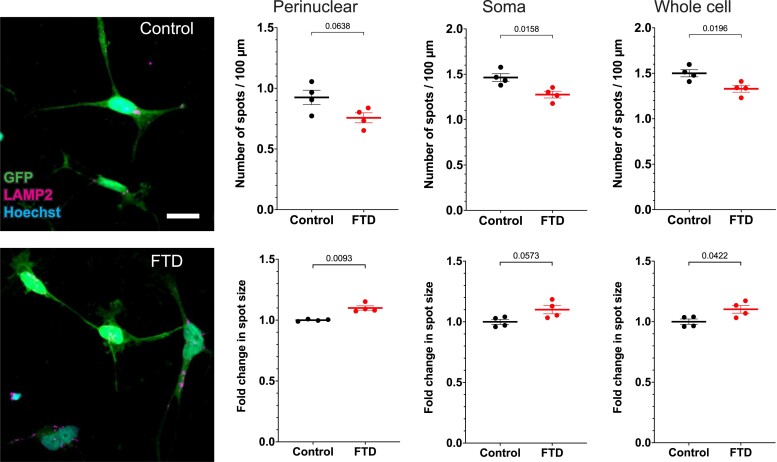
**Alterations of the endolysosomal system in co-cultures of FTD neurons and astrocytes.** Immunocytochemistry analysis of LAMP2 in GFP-labelled iAs derived from Control and FTD-iPSC lines, and comparison of the number of dots and change in dot size in the perinuclear and somatic areas as well as in the whole cell. Data are presented as mean ± SEM from four independent experiments. Each experiment included between 111 and 805 cells, with a total of 1767 Control-iAs and 1165 FTD-iAs. A two-tailed *t*-test with Welch’s correction was used to analyze significance. *P-*value is shown in the graph for each comparison. Scale bar = 50 µm.

Given the important role of CHMP2B in the endolysosomal pathway, it is highly relevant to investigate alterations of this system in FTD co-cultures. To do so, we carried out immunocytochemistry of markers for early endosomes (EEA1), late endosomes/lysosomes (LAMP2), and aggregates (p62) in both iNs and iAs in co-culture after 42 days of differentiation, and quantified the number and size of puncta in different regions of the cell using the Operetta confocal microscopy high-content screening system (Supplementary Fig. 3). Our results showed that FTD-iAs displayed decreased number but increased size of LAMP2 puncta (Fig. 4), suggesting impairment of the endolysosomal system specifically in astrocytes. On the other hand, we could not detect any clear alterations in early endosomes or aggregates in FTD-iAs, or in any marker in FTD-iNs (Supplementary Fig. 4A, B). These results indicate that the first clear impairments are in late endosomes/lysosomes and occur in astrocytes before protein aggregation, as expected by dysfunction of CHMP2B and the ESCRT-III complex.

Altogether, our results suggest that the intron 5 mutation in the *CHMP2B* gene does not impair differentiation towards iNs and iAs, and that synapse numbers are not affected. However, a striking reduction of CHMP2B protein, slight alterations in neuronal arborization of FTD-iN, as well as in the endolysosomal system of FTD-iAs, were evident. These findings confirm that our model and co-culture system recapitulate FTD phenotypes, revealed previously unknown alterations in FTD astrocytes, and can be useful to identify and study disease mechanisms in different brain cells.

### CHMP2B mutation does not trigger alterations in neuronal intrinsic properties or synaptic currents

**Figure 5:**
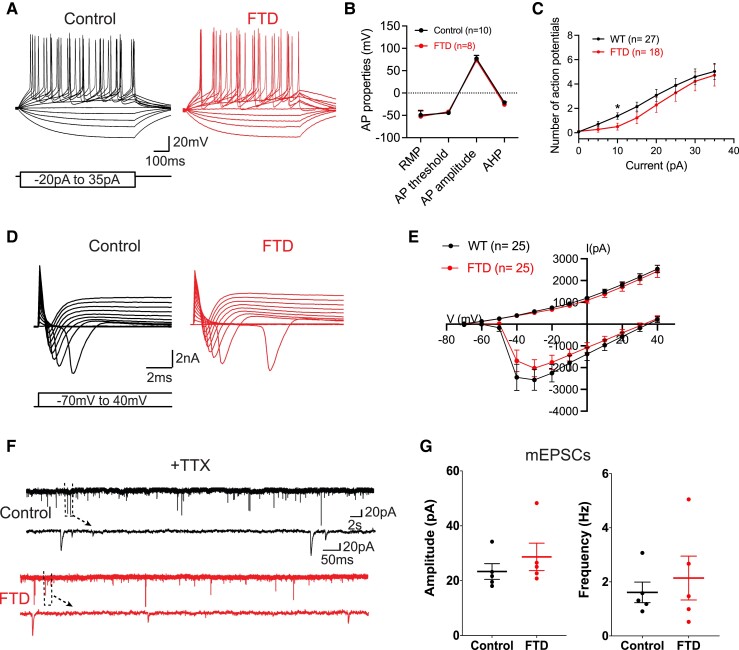
**Control- and FTD-iNs are functional. (A)** Control- and FTD-iNs both generate multiple action potentials (APs) upon stepwise current injection in 5 pA increments, from holding potential of -70 mV. **(B)** AP parameters in iNs. The total *n* indicated in the figure comes from three independent experiments with 2–4 cells each. **(C)** Cumulative number of APs from depolarization steps (shown in A). **(D)** Typical inward sodium and outward potassium currents in Control- and FTD-iNs upon application of depolarizing steps of 10 mV from -70 mV holding potential. **(E)***I*/*V* curves of inward and outward currents illustrated in D. **(F)** Example traces with magnified insets showing miniature excitatory postsynaptic currents (mEPSCs) in the two iN groups, recorded in presence of TTX. **(G)** Amplitude and frequency of recorded mEPSCs in the two groups. Data are presented as mean ± SEM of the neurons indicated in each graph generated in three independent experiments. A two-tailed Mann–Whitney test (**C**, **G,** and **F**) or multiple *t*-test (**D** and **E**) was used to analyze significance. *P ≤ 0.05.

Considering that neuronal functionality has not yet been investigated in human cells with mutations in *CHMP2B*, we performed whole-cell patch-clamp recordings to investigate alterations in the electrophysiological properties of FTD-iNs in co-culture at 40–50 days of differentiation. First, we examined passive membrane properties but did not find differences in membrane capacitance, Ri, or the RMP (Supplementary Fig. 5A). Next, we investigated excitability, showing that both Control- and FTD-iNs were generating APs with similar characteristics (Fig. 5A-C) and equally expressing fast inward sodium and sustained outward potassium currents (Fig. 5D and E).

We then sought to investigate if iNs could form synaptic connections and whether functional capacity was affected in the FTD line. We identified excitatory postsynaptic currents in both Control- and FTD-iNs and burst-like activity typical for excitatory neurons co-cultured with astrocytes (Supplementary Fig. 5B, C). To objectively evaluate synaptic function, we analyzed mEPSCs in the presence of sodium channel blocker, TTX, which eliminated bursting activity and allowed us to better appreciate parameters of detected synaptic currents (Fig. 5F). We did not see any difference either in frequency or amplitude of mEPSCs (Fig. 5G), nor did we see any difference in maximum amplitude, bursts, burst duration, or frequency of synaptic events (Supplementary Fig. 5D).

Therefore, we concluded that our co-culture system is useful for studying neuronal electrophysiological properties and that the intron 5 mutation did not cause any major functional alterations detectable at either individual iN or synapse level.

### CHMP2B mutation alters astrocyte glutamate homeostasis and calcium signaling

**Figure 6:**
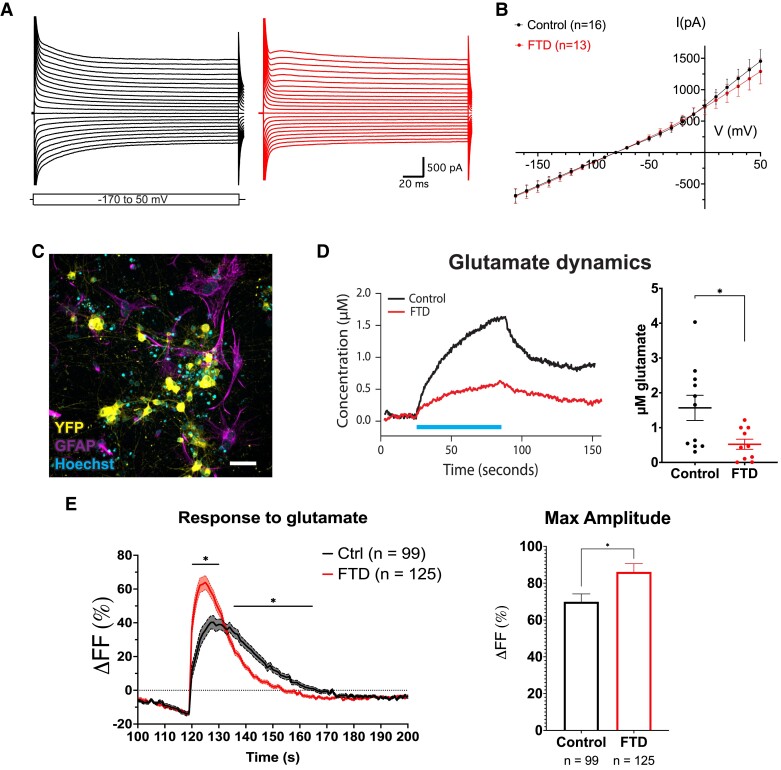
**Control- and FTD-iAs present potassium currents and FTD-iAs display increased capacity taking up glutamate and sensing extracellular glutamate**. (**A**) Current traces illustrating typical potassium currents activated in iAs during voltage steps ranging from -170 to +50 mV in 10 mV steps. (**B**) *I*/*V* curve of quantified currents induced by voltage steps. Data are presented as mean ± SEM of the indicated number of cells from three independent experiments. A multiple *t*-test was used to analyze significance. **(C)** Immunocytochemistry of a representative co-culture of neurons and astrocytes after lentiviral transduction with ChR2-YFP. **(D)** Representative traces showing increase in glutamate after optogenetic stimulation of neurons in co-culture (left graph) and average increase in glutamate concentration of Control and FTD co-cultures after stimulation (right graph). Data are presented as mean ± SEM from 11 (Control) and 10 (FTD) independent cultures from four differentiations. A two-tailed *t*-test with Welch’s correction was used to analyze significance. *P ≤ 0.05. **(E)** Average traces showing increase in intracellular calcium measured as fluorescence change after glutamate stimulation in iAs (left graph) and maximum amplitude in intracellular calcium measured as fluorescence change of Control and FTD iAs after glutamate stimulation (right graph). Graph shows seconds 100 to 200 of a 300 s recording with glutamate being administered at second 120 of the recording. Data are presented as mean ± SEM from the indicated number of cells derived from two to four cultures from two independent differentiations. A multiple *t*-test (left graph) and a two-tailed *t*-test with Welch’s correction (right graph) were used to analyze significance. *P ≤ 0.05. Scale bar = 50 µm (**C**).

Next, we investigated electrophysiological properties of iAs in monoculture. We did not find any differences in either passive membrane properties (Supplementary Fig. 6A, B) or potassium currents (Fig. 6A and B) in FTD-iAs as compared to Control-iAs.

An essential astrocyte function altered in several neurological disorders is the uptake of neurotransmitters released by neurons into the synaptic cleft. In addition, a previous study on FTD described increased astrocytic glutamate uptake.^15^ To investigate this property in iAs in co-culture with iNs, we used an optogenetic approach combined with electrochemical detection of glutamate (Fig. 6C). This experimental setting allowed us to stimulate release of glutamate from iNs in the co-culture while quantifying its increase in the medium, giving an indication of astrocytic uptake dynamics (Fig. 6D). Results showed that upon stimulation, in Control co-cultures, the peak of glutamate detected in the medium was three times higher than the one in FTD co-cultures (Control 1.57 ± 0.36 versus FTD 0.52 ± 0.14). Since synaptic recordings did not reveal any difference in release of glutamate between FTD and control neurons (Fig. 5G), this result suggests that more glutamate is being taken up by FTD-iAs. To confirm that glutamate was taken up through glutamate transporters, we performed a glutamate uptake assay in iAs monocultures 42 days after induction with or without DL-threo-β-benzyloxyaspartic acid (DL-TBOA) in the medium, a specific blocker of GLT1 and GLAST, showing that in the presence of the blocker, iAs were not capable of taking up any glutamate compared with cultures without DL-TBOA (Supplementary Fig. 6C). Interestingly, glutamate and glutamine content in FTD-iNs and -iAs was similar to Control-iNs and -iAs (Supplementary Fig. 6D), indicating that the glutamate–glutamine cycle was not altered and suggesting that the alteration in glutamate uptake was likely due to an increased capacity to take up glutamate by FTD-iAs. We next analyzed expression levels of glutamate transporters at 21 days of differentiation in isolated iAs, which already displayed increased glutamate uptake. Our results showed that FTD-iAs have similar expression levels of both *GLT1* and *GLAST* compared to Control-iAs, suggesting that differences in glutamate uptake were not due to an increased expression levels of glutamate transporter mRNA in FTD-iAs (Supplementary Fig. 6E).

In addition to take up neurotransmitters, astrocytes also have glutamate receptors that allow them to detect and respond to increases in glutamate at the synaptic cleft.^36^ It has been proposed that glutamate stimulation of astrocytes can drive the release of gliotransmitters, regulating neuronal and network functionality.^37^ Therefore, we decided to interrogate this important function of astrocytes using live cell calcium imaging of iAs in monocultures at 42–49 days of differentiation following glutamate stimulation. Results showed a striking differential response of Control- and FTD-iAs to glutamate, with FTD astrocytes presenting an increased maximum amplitude after glutamate stimulation and a faster recovery to normal intracellular calcium levels (Fig. 6E).

Altogether, our data indicate that *CHMP2B* mutation does not affect electrophysiological properties of iAs, but alters the response to glutamate with increased uptake and responsiveness resulting in aberrent calcium signalling.

### FTD presents alterations in the connectivity structure of neuronal networks

Changes in synaptic glutamate homeostasis and astrocytic response to glutamate, can lead to alterations in neuronal networks. To investigate potential connectivity abnormalities in FTD networks, we carried out calcium imaging assays using Fluo-4 calcium indicator coupled with network activity analysis in co-cultures of iNs and iAs at 47–49 days of differentiation. Although we detected mainly iNs loaded with Fluo-4, we separated iNs and iAs based on their spike dynamics, with neuronal spikes being faster than astrocytic. We recorded spontaneous activity and response of iNs to K^+^ stimulation (Supplementary Fig. 7A), however, there were no significant differences between Control and FTD (Supplementary Fig. 7B), confirming that iAs potassium buffering was not impaired.

**Figure 7:**
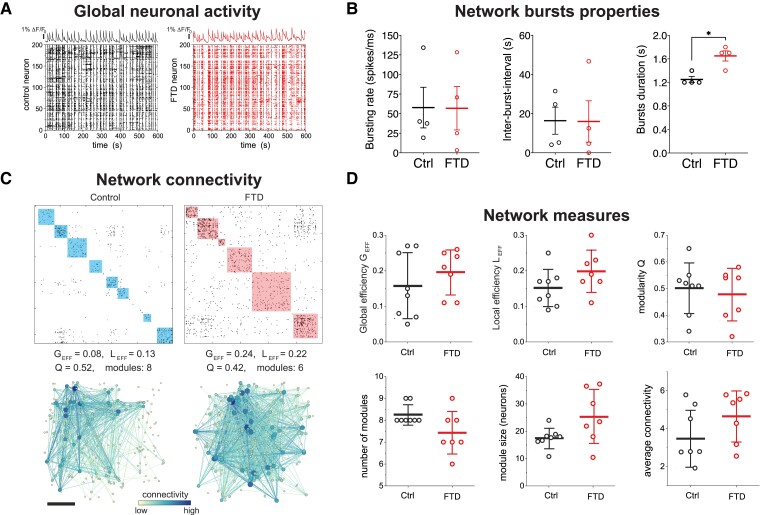
**Neuronal network alterations in FTD co-cultures.** (**A**) Representative data of spontaneous activity in control (left) and FTD (right) cultures. Top panels show average fluorescence traces of neurons, with sharp peaks signaling strong collective activity (network bursts), while bottom panels show corresponding raster plots with columns representing time and rows representing individual neurons. (**B**) Different indicators for network bursting characteristics. Both cultures have similar behavior, but FTD exhibits significantly longer bursts, i.e. higher number of spikes are elicited upon neuronal activation in FTD as compared to controls. Data are presented as mean ± SEM from four independent experiments. A two-tailed *t*-test with Welch’s correction was used to analyze significance. *P ≤ 0.05. (**C**) Top panels show representative functional connectivity matrices of Control and FTD co-cultures, where black dots are functional connections (significant interactions) among neuronal pairs and colored boxes are functional modules, which reflect the tendency of neurons to communicate within small groups. Bottom panels show the corresponding network maps. (**D**) Graphs showing results of functional analysis of neuronal networks comparing Control and FTD co-cultures. Analysis include Global Efficiency, Local Efficiency, Modularity *Q*, number of modules per culture, number of neurons per module, and average network connectivity. Data are presented as mean ± SEM from seven to eight independent cultures from four different experiments. A two-tailed *t*-test with Welch’s correction was used to analyze significance. Ctrl = Control.

Given the alteration observed in FTD-iAs and the role of astrocytes in regulating neuronal function, we next proceeded to examine spontaneous activity of individual iNs as well as neuronal network activity. We detected clear global network activity with continuous network bursting events, which are simultaneous firings of many iNs comprising the circuit (Fig. 7A). We did not find any difference in firing rate, ISI, or the percentage of iNs participating in network bursts (Supplementary Fig. 7C) nor in any traits of the bursts that we analyzed at the individual neuronal level (Supplementary Fig. 7D). Thus, we concluded that individual neuronal activity was not affected. We next examined neuronal network properties and connectivity, finding that global network bursting activity, IBI, firing activity, and mean spikes were similar in both Control and FTD (Fig. 7B and Supplementary Fig. 7E). However, burst duration was clearly different, with FTD-iNs displaying longer bursts than Control-iNs (Fig. 7B). Additionally, and although both Control and FTD neuronal networks exhibited a very similar behavior with whole-network activations in the form of bursts, there was a striking difference in the structure of the network functional modules, which are groups of iNs that communicate within the group more strongly than with the rest of the network (Fig. 7C). Clearly, the Control network exhibited more functional modules, of smaller size, and importantly, more isolated, as revealed by the density of connections outside groups. These connections reflect the interconnection among modules and overall synchronization of the network. A high density of connections, as occurs for FTD, indicates that the network tends to activate in a strongly synchronous manner, while a low density indicates that there is a balance between communication at a local (modules) and global level (whole network).

To in-depth examine these differences, we investigated functional connectivity of neuronal circuits extracting several descriptors that capture their functional organization. These descriptors, termed ‘network measures’ quantify aspects that allow us to define whether a network is segregated and forming different isolated modules or integrated and organized in interconnected modules (Supplementary Fig. 7F). The Global Efficiency *G*_EFF_ reflects tendency for global communication across the network and within modules, which was lower for Control than for FTD (Fig. 7D), confirming the idea that FTD networks have more connected modules. The Local Efficiency *L*_EFF_ captures strength of functional coupling within modules, which is also lower for Control and indicates that neurons within the modules are more strongly connected in FTD networks. The modularity *Q* indicates how strong the functional modules are. The smaller, more abundant, and more disconnected the modules, the higher *Q*, which is the case for the Control network and indicates a more refined global network organization. This is confirmed by the higher number of modules and their smaller size considering the number of iNs within modules, which were also quantified (Fig. 7D). The number of functional connections for FTD was higher than the Control, consistent with higher density of links in the connectivity matrices for the former, as shown by the average connectivity measure (Fig. 7D). Strikingly, these measures indicate that the Control network balances global and local communication, while the FTD network tends to excessively integrate the circuit. Functional connections for Control are more localized, preferentially involving nearby iNs, while FTD connections tend to extend the entire area of the culture. On average, overall data shows a tendency for FTD co-cultures to shape more synchronous networks than the Control co-cultures, with a much higher integration (high *G*_EFF_ and *L*_EFF_) and weak functional module formation (low *Q*, less abundant and bigger modules).

In summary, our data indicates that the *CHMP2B* intron 5 mutation does not affect activity patterns of individual neurons but there is a significant effect at the neuronal network connectivity level, which is illustrated by an increased synchronization of FTD networks. This increase is represented by elevated number of neurons within each functional module and the number of connections between different functional modules, increasing the overall average connectivity and synchronicity of the network.

## Discussion

Here, we present a new hESC-based model of FTD caused by a mutation in the *CHMP2B* gene. Using CRISPR/Cas9 genome editing we generated a hESC line carrying the intron 5 mutation and differentiated hESCs to iNs and iAs using transcription factor programming. We co-cultured these two cell types together to create a neuron-astrocyte model that allowed us to identify an alteration in the endolysosomal system of FTD astrocytes. Furthermore, we have shown impairments in astrocytic functions related to glutamate homeostasis and importantly, altered connectivity and structure of FTD neuronal networks.

By selecting efficient sgRNAs, designing ssODNs with short homology arms and phosphorothioate bonds, and using both WT and mutated ssODNs, we obtained HDR efficiencies above 10% and generated several heterozygous clones. This was key since patients with *CHMP2B* mutation are heterozygous. This approach facilitates generation of human disease-specific pluripotent stem cell lines using CRISPR/Cas9. In combination with the parental line, our isogenic FTD-hESC pair represents a very valuable resource for studying FTD.

Our new hESC-derived co-culture system is based on transcription factor programming protocols that allow for specific and independent generation of excitatory neurons and astrocytes with high efficiency and purity. The first and most important advantage of these protocols is the purity and fast maturation of cells, allowing for studies focused on pathophysiology of neurodegenerative disorders that do not affect development. Second, with this approach, both cell types can be studied independently or in co-cultures in which the ratio of each cell type can be controlled. We anticipate this technique to be very useful for modeling neurodegenerative disorders.

To date, most studies on FTD caused by *CHMP2B* mutations have been performed in murine models, however, neurodegeneration in animal models is not always a good correlate to the human disease.^38^ Moreover, these models have been mainly generated by overexpression of the mutated forms, while human patients have one healthy and one mutated allele. Here, we show that despite the presence of the mutation, the levels of total mRNA from the *CHMP2B* gene are not decreased, although we could not differentiate the contribution of each allele, and from the two described isoforms originating from the intron 5 allele, only one was detected here. Interestingly, when analyzing protein levels in iNs and iAs independently, we found a striking decrease in both cell types, going beyond what would be expected from a heterozygous loss. In addition, we could not detect any truncated isoform, although we cannot rule out that this is due to inefficient binding of the antibody to the truncated protein. Regardless, our data on mRNA and protein levels suggest that animal models based on overexpression of truncated CHMP2B isoforms are not representative of human physiological conditions and highlight the importance of using human models with normal levels of *CHMP2B* expression.

Recently, a patient-derived iPSC model has been used to investigate disease mechanisms at a stage in which endolysosomal alterations leading to aggregation were evident.^14^ However, there is a lack of studies showing functional alterations. In our study, we have generated neuron-astrocyte co-cultures that present a slight but consistent decrease in number and increase in size of LAMP2+ vesicles (late endosomes and lysosomes) in astrocytes. A change in late endosomes and lysosomes is a hallmark of FTD caused by *CHMP2B* mutations, however, to date, most studies have been focusing on neurons, and our results suggest that astrocytes also present alterations in the endolysosomal system.

Interestingly, these alterations do not affect electrophysiological properties at the individual level in astrocytes but potentially affect their glutamate uptake capacity and their response to extracellular glutamate. A hypothetical explanation is an abnormal recycling of glutamate transporters and receptors in the membrane of astrocytes, a process that is dependent on endosomes and the ESCRT-III complex, which could lead to increase in glutamate transporters and receptors at the synapse. These results are in line with a previous report on *CHMP2B*-FTD iPSC-derived astrocytes, which showed that glutamate uptake was increased in disease astrocytes.^15^ The importance and abundance of these proteins in the astrocyte membrane could make them more sensitive to endolysosomal alterations and produce this phenotype.

An important result from our study is that FTD neuronal networks burst more strongly, suggesting that there is excess excitability in FTD circuits. Considering that our co-culture system only contains excitatory neurons, there is no difference in amount of inhibition between Control and FTD. Thus, a possible explanation for the excessive excitability in FTD networks is that neurons form stronger functional coupling as compared to Controls. We must note that network analyses are extracted from activity of the iNs and not from the underpinned physiological circuit organization, which is unknown. Translating genetic anomalies of FTD-iNs to structural deficits and functional alterations is not straightforward since the development of the circuit and plasticity mechanisms along maturation can play an important role in shaping functional traits. The higher synchronization of FTD networks suggest that regulatory mechanisms are not operating correctly. Excessive synchronization of neuronal circuits *in vitro* due to genetic disease has also been observed in a study showing a tendency towards synchronous dynamics and excessive integration in Parkinson disease patient-derived iPSC neuronal cultures ascribed to the gradual loss of dopaminergic neurons in the circuit.^39^ As a potential explanation for these phenotypes, we hypothesize that the increased calcium signaling in astrocytes following the higher response to extracellular glutamate could lead to an excessive release of gliotransmitters and hypothetically cause the neuronal network hyperexcitability and hypersynchronization. Strikingly, these functional phenotypes arise after small alterations in the endolysosomal system, suggesting that membrane proteins essential for proper functionality of brain cells are highly susceptible to endolysosomal dysfunction. Our results imply that astrocyte dysfunction could precede neuronal alterations in FTD and that these impairments could induce alterations to neuronal network function. This can have important consequences for studies of other proteinopathies and highlights the importance of examining astrocytes in neurodegenerative diseases. In addition, it suggests the possibility to use transcranial magnetic stimulation to detect early circuit alterations as a future option for early diagnosis of neurodegenerative disorders. We anticipate that our co-culture system will be very useful not only in *CHMP2B*-FTD, but also in other neurological disorders in which the role of astrocytes is starting to be recognized.^40^ The possibility to independently and combinedly examine different brain cells provides the field with a unique tool for the study of cell type-specific contributions to neurological disorders.

In conclusion, we have shown that combining CRISPR/Cas9 to introduce disease-causative mutations in pluripotent stem cells with transcription factor programming to neurons and astrocytes represents an efficient strategy to model neurodegenerative diseases. This approach revealed functional alterations in astrocytes related to glutamate and alterations in the neuronal network, thereby, contributing to FTD pathogenesis. Furthermore, we propose that functional connectivity alterations in form of hyperactivity and high synchronicity is a relevant phenotype in *CHMP2B*-FTD and possibly other human neurodegenerative disorders.

## Supplementary Material

fcad158_Supplementary_Data

## Abbreviations

aCSF =: artificial cerebrospinal fluid
AHP =: after hyperpolarisation
AM =: acetoxymethyl ester
AP =: action potential
BSA =: bovine serum albumin
B-III-tub =: beta-III-tubulin
Cas9 =: CRISPR associated protein 9
CRISPR =: clustered regularly interspaced short palindromic repeats
Ctrl =: control
D-APV =: D-2-amino-5-phosphonovalerate
DL-TBOA =: DL-threo-β-benzyloxyaspartic acid
DMSO =: dimethyl sulfoxide
Dox =: doxycycline
EPSC =: excitatory postsynaptic current
ESCRT-III =: endosomal sorting complex required for transport III
FOV =: field of view
FPS =: frames per second
FTD =: frontotemporal dementia
GFAP =: glial fibrillary acidic protein
GFP =: green fluorescent protein
GluOX =: L-glutamate oxidase
GNA =: global network activity
GTE =: generalized transfer entropy
HDR =: homology-directed repair
hESC =: human embryonic stem cell
hygro =: hygromycin
iAs =: induced astrocytes
IBI =: inter-burst-interval
iNs =: induced neurons
iPSC =: induced pluripotent stem cell
IRES =: internal ribosome entry site
ISI =: inter-spike interval
LAMP2 =: lysosome-associated membrane protein 2
LED =: light-emitting diode
MEAs =: multielectrode arrays
mEPSC =: miniature excitatory postsynaptic current
NBQX =: 2,3-dioxo-6-nitro-1,2,3,4-tetrahydrobenzo[*f*]quinoxaline-7-sulfonamide
Nfib =: nuclear factor I B
Ngn2 =: neurogenin 2
PAM =: protospacer adjacent motif
puro =: puromycin
qPCR =: quantitative PCR
Ri =: input resistance
RMP =: resting membrane potential
ROCK =: rho-associated coiled-coil containing protein kinase
ROI =: region of interest
rtTA =: reverse tetracycline transcriptional activator
SEM =: standard error of the mean
sgRNA =: single-guide RNA
shRNA =: short hairpin RNA
Sox9 =: SRY-Box transcription factor 9
ssODN =: single-stranded oligodeoxynucleotide
T2A =: thosea asigna virus 2A sequence
tetO =: tetracycline operator
TTX =: tetrodotoxin
Ubiq =: ubiquitin
WT =: wild type
YFP =: yellow fluorescent protein
