# Supplementary material for: Astrocyte dysfunction and neuronal network hyperactivity in a CRISPR engineered pluripotent stem cell model of frontotemporal dementia
Source: Brain Commun. 2023 May 18;5(3):fcad158. doi: 10.1093/braincomms/fcad158 (PMC10233896; doi:10.1093/braincomms/fcad158)
Supplement: fcad158_Supplementary_Data [file fcad158_supplementary_data.docx]

**Supplementary materials and methods**

*Cell lines*

H1 (WA01) hESCs from WiCell Research Institute (WiCell, WI) were cultured in feeder-free conditions in mTeSR1 medium (StemCell Technologies) on Matrigel-coated six-well plates (Corning), with full medium changes daily. When reaching ~80% confluency, cells were dissociated with Accutase (Thermo Fisher Scientific) and 125.000-200.000 cells replated in new Matrigel-coated 6-well plates with mTeSR1 medium supplemented with 500 nM thiazovivin (Sigma-Aldrich) during the first 24 h.

*Karyotyping of hESC lines*

At approximately 80% confluency, cells were incubated at 37˚C for 40 min in fresh mTeSR1 containing KaryoMax Colcemid at a concentration of 20 ng/ml. Cells were then washed twice with PBS, followed by dissociation with Accutase into a single cell suspension. Cells were collected with mTeSR1 medium, transferred into a 15 ml-tube, and centrifuged at 200 g for 5 min at room temperature (RT). After aspiration of the supernatant, cells were washed with 5 ml of DPBS and centrifuged at 1200 rpm for 5 min at RT. Following aspiration of the supernatant, the pellet was vortexed at low speed while 10 ml of 0.075 M KaryoMax KCl prewarmed at 37°C was added drop by drop and incubated for 10 min at 37˚C. Subsequently, 1 ml of freshly prepared cold (-20˚C) Carnoy fixative (methanol:acetic acid, 3:1) was added drop by drop. Samples were centrifuged at 300 g for 10 min at RT, supernatants aspirated, and pellets resuspended in 10 ml of cold Carnoy fixative added drop by drop while vortexing at low speed. Samples were stored at -20˚C until sent for karyotyping analysis by Ambar Anàlisis Mèdiques (Barcelona).

*Real-Time qPCR*

RNA isolation was carried out using the Rneasy Mini Kit (Qiagen) according to the manufacturer’s instructions and treating RNA with Dnase I (Qiagen) to prevent DNA contamination. RNA concentration was determined using a Nanodrop ND-1000 spectrophotometer (Saveen & Werner). One microgram of RNA was used in a reverse transcription reaction using the qScript cDNA Synthesis Kit (Quantabio). Real-time qPCRs were carried out with TaqMan Universal PCR Master Mix and TaqMan assays (Supplementary Table 4) on an iQ5 Real-Time PCR detection system (Bio-Rad).

*Cell death analysis*

Control- and FTD-hESC lines were collected at day 2 after split, by a 5-min incubation with Accutase and apoptosis was assessed using the BD Pharmingen Apoptosis detection kit (BD Biosciences) according to manufacturer’s instructions. Briefly, the cells were washed with PBS and resuspended in Binding Buffer before being stained with Annexin V and 7-AAD for 15 min at RT. Annexin V and 7-AAD stainings, as a measure of apoptosis, were detected by flow cytometry using a BD LSR Fortessa.

*Proliferation analysis*

At day 2 after split, Control- and FTD-hESC lines were assessed using the Click-iT EdU Alexa Fluor 647 Flow Cytometry Assay Kit (Thermo Fisher Scientific) according to manufacturer’s instructions. Briefly, the cells were pulsed with 5µM EdU for 30 min at 37°C and collected by a 5-min incubation with Accutase. Next, the cells were fixed and permeabilized and EdU was detected by flow cytometry using a BD LSR Fortessa.

*Immunocytochemistry*

All primary and secondary antibodies used in this study can be found in Supplementary Tables 5 and 6. At indicated times, cells grown on coverslips were washed once with potassium-phosphate-buffered saline (KPBS) and fixed for 15 min at RT in 4% paraformaldehyde (PFA), washed once with KPBS and blocked for 60 min in KPBS containing 0.025% Triton X-100 (TKPBS) and 5% normal donkey serum (NDS; Millipore). Primary antibody incubation was carried out overnight at 4 °C in blocking solution. Cells were then washed twice for 5 min with 0.025% TKPBS and once for 5 min with blocking solution. Incubation with secondary antibodies was performed in blocking solution for 2 h at RT. Cells were then washed once with 0.025% TKPBS for 5 min and twice for 5 min with KPBS, and coverslips were mounted using PVA:DABCO, polyvinyl alcohol (Sigma-Aldrich)-based mounting media containing DABCO (Sigma- Aldrich) anti-fading reagent. For nuclear staining, 1 µg/ml of Hoechst (Thermo Fisher Scientific) was used during the first washing step after the secondary antibody incubation. Images were obtained with a Zeiss LSM 780 confocal microscope and orthogonal reconstructions were done with Zen software.

*Cell lysis and western blot*

Cocultures of iN and iAs were grown in 6-well plates with 413.000 iN plated on inserts and 332.000 iAs plated on the wells, both coated with Matrigel. On day 42 of differentiation, cells were washed twice with cold PBS on ice. Cell from 2-3 wells per experimental condition were collected by pipetting (iNs) or scrapping (iAs) in accutase, followed by centrifugation and the supernatant discarded. Cells pellets were snap frozen and kept at -80^o^C. Pellets corresponding to 8 x10^5^ (iNs) or 6 x10^5^ (iAs) cells were lysed in 50 mM Tris–HCl pH 7.5, 0.1 mM EDTA, 0.1 mM EGTA, 0.1 mM β-mercaptoethanol, containing 0.5% (w/v) SDS, 0.1 mM sodium orthovanadate and protease inhibitors (2 μg/ml each of leupeptin, aprotinin and trypsin inhibitor, and 1.3 mM Pefablock) by forced passes through a 26 1/2G needle. Protein concentration in homogenates was determined by the bicinchoninic acid (BCA) method (Thermo). Lysates were denatured at 95ºC for 5 min in Laemmli buffer and aliquots containing 25 μg of protein were separated on 12.5% (w/v) SDS-polyacrylamide gels. Proteins were transferred to Immobilon-P membranes (Millipore) using a semi-dry transfer system from Bio-Rad. Blots were blocked with 2% (w/v) powdered skimmed milk in T-TBS (20 mM Tris-HCl, pH 7.5, 500 mM NaCl, 0.05% (v/v) Tween-20), before incubation with the antibodies of interest diluted in 1% (w/v) BSA in T-TBS. The primary antibody used was anti-CHMP2B followed by the corresponding HRP-conjugated secondary antibody (1:2000 v/v, Dako). Bands of interest were visualized by chemiluminiscence using the ECL system (Cytiva), exposing blots to X-Ray film (Agfa). For normalization the membranes were stripped and stained with Ponceau. Membranes were thoroughly washed with water before stripping with 8 M guanidinium chloride pH 3 for 30s. Blots were then washed again with water and then stained with Ponceau 1% (w/v) in 1% (v/v) acetic acid for 15 min.

*Organelle quantifications with Operetta confocal microscope*

High content screening was performed by imaging prepared immunofluorescence samples using the automated Operetta CLS confocal microscope (PerkinElmer) with a 40x high NA water immersion objective for high spatial resolution images. Forty individual, non-adjacent fields were acquired per experimental sample, with each field consisting of 3x z planes at a 1.5 µm step. The z stack for each field was processed in Harmony (PerkinElmer) as a maximum projection, and an analysis pipeline was developed to identify neuronal and astrocytic cells, but to discard those cells forming groups of >4 cells prior to data analysis. Cell nuclei were identified using Hoescht localisation, with neurons (MAP2+ cells) and astrocytes (GFP+ cells after induction with a tetO.GFP virus during the induction step) being specified using a threshold acting upon the mean cytoplasmic intensity across each cell. Cell subregions (nuclei, perinuclear region, soma, whole cell) were specified based on nuclear and cytoplasmic intensity and spot detection was performed in each region.

*Sholl analysis and synaptic quantification*

Co-cultures on coverslips were generated as described above. For each coverslip, 50-100 iNs infected with tetO.GFP lentivirus were plated together with unlabeled iNs. For sholl analysis, images of neurons were acquired with a Zeiss LSM 780 confocal microscope with a 10x objective lens and analyzed with Zen software. 3-5 neurons per coverslip were analyzed from a total of eight coverslips from four individual experiments for each sample. Branches were quantified using the Fiji Sholl analysis plugin^1^. For quantification of Bassoon-PSD95 co-staining, images of neurons were acquired with a Zeiss LSM 780 confocal microscope with a 63x objective lens. Laser power and photomultiplier gain were set at the same levels for all the samples to allow for quantitative comparisons. Five neurons per coverslip were analyzed from a total of eight coverslips from four individual experiments for each sample. The GFP-labelled region was defined using the threshold option in Fiji and colocalizing puncta inside the GFP-labelled region were detected with the colocalization threshold plug-in of Fiji. We quantified the number of puncta inside the GFP region by using the particle analysis plug-in of ImageJ and counting puncta >0.1 µm in size to discard background.

*Glutamate uptake*

iAs were plated on day 7 on Matrigel-coated 4-well plates and cultured until day 42. Cells were preincubated 5 min with Hank’s balanced salt solution (HBSS++, Gibco) with or without DL-TBOA (Tocris Bioscience), and later incubated for 5 min in HBSS++ containing 200 μM glutamate with or without DL-TBOA. Samples of medium were collected after 5 min and analysed with a fluorometric glutamate assay kit (Abcam) according to the manufacturer’s instructions.

*Glutamate and glutamine quantification*

Co-cultures of iNs/iAs using inserts (iNs on the insert and iAs on the well) were dissociated with Accutase and pellets of iNs or iAs were resuspended in DPBS and used to quantify the amount of protein using the Pierce Coomassie Plus Bradford Assay Kit (Thermo Fisher Scientific) following the manufacturer’s instructions, and the cell glutamine and glutamate content was quantified using the Glutamine/Glutamate-Glo Assay (Promega) following the manufacturer’s instructions.

References

1. Ferreira TA, Blackman AV, Oyrer J*, et al*. Neuronal morphometry directly from bitmap images. *Nat Methods*. Oct 2014;11(10):982-4. doi:10.1038/nmeth.3125

**Supplementary Table 1. CHMP2B genome editing oligonucleotides**

| **Name** | **Sequence*** |
| --- | --- |
| sgRNA 4 | 5’-TCGAGCAGCTGATGGAGCTT-*TGG*-3’ |
| WT donor strand | 5’-TAATGCACGTTTGTCTTTTTCATTGTTTAATATAGATGG  C**T**AAAGCTCCATCAGCTGCTCGAAGCTTACCATCTG-3’ |
| I5 mutation donor strand | 5’-TAATGCACGTTTGTCTTTTTCATTGTTTAATATACATGG  C**T**AAAGCTCCATCAGCTGCTCGAAGCTTACCATCTG-3’ |

*The change in the PAM sequence of the donor strands is marked in blue while the mutation is marked in red and the WT nucleotide in the position where the mutation is located is marked in green.

**Supplementary Table 2. Primers for CHMP2B and off-target genomic sites**

| **Genomic loci** | **Primer** | **Sequence** |
| --- | --- | --- |
| CHMP2B | Forward | 5’-AGTTTCTGCCTACCACGTTTG-3’ |
|  | Reverse | 5’-AAAGCCTTGAGTTGCCGTTC-3’ |
| Off-target Chr 1 | Forward | 5’-GTAAAAGCTGCACGCCAACT-3’ |
|  | Reverse | 5’-TGAATGCCCAGACTCTCCTC-3’ |
| Off-target Chr 7 | Forward | 5’-AGAGGCCATCACCTTCCAG-3’ |
|  | Reverse | 5’-CATCCGTTTGGTCCTGATG-3’ |
| Off-target Chr 10 | Forward | 5’-GTTGCTCCGGCTTTCAGAT-3’ |
|  | Reverse | 5’-GCCAGACCAAGTGTTTGGAT-3’ |
| Off-target Chr 15 | Forward | 5’-GGCTTCCAGTTGCTTGAAAA-3’ |
|  | Reverse | 5’-GAAACCGACTTCTATCTCTCCTC-3’ |
| Off-target Chr 17 | Forward | 5’-CTCTGCGTCACCCACTGT-3’ |
|  | Reverse | 5’-ACTTCTCGTACCTGTCCCAA-3’ |

**Supplementary Table 3. Lentiviral vectors**

| **Viral vector** | **Addgene catalog number** |
| --- | --- |
| pMDLg/pRRE | #12251 |
| pMD2.G | #12259 |
| pRSV-Rev | #12253 |
| M2-rtTA | #20342 |
| tetO-FUW-GFP | #30130 |
| tetO.Sox9.Puro | #117269 |
| tetO.Nfib.Hygro | #117271 |
| pTet-O-Ngn2-puro | #52047 |
| pLenti-CaMKIIa- hChR2(E123T/T159C)-EYFP-WPRE | Duke Viral Vector Core |

**Supplementary Table 4. TaqMan assays**

| **Gene name** | **Assay ID** |
| --- | --- |
| GAPDH | Hs02758991_g1 |
| CHMP2B | Hs01045897_m1 |
| S100B | Hs00902901_m1 |
| MAP2 | Hs00258900_m1 |
| GLAST | Hs00188193_m1 |
| GLT1 | Hs01102423_m1 |
| NANOG | Hs02387400_g1 |
| OCT3/4 | Hs00742896_s1 |

**Supplementary Table 5. Primary antibodies**

| **Antibody** | **Vendor** | **Catalog number** | **Dilution** |
| --- | --- | --- | --- |
| rabbit α-Beta-III-tub | Biolegend | 802001 | 1:1000 |
| chicken α-MAP2 | Abcam | ab5302 | 1:5000 |
| guinea pig α-GFAP | Synaptic systems | 173004 | 1:500 |
| goat α-GFP | Abcam | Ab5450 | 1:2000 |
| mouse α-LAMP2 | Hybrydoma Bank | H4B4 | 1:200 |
| mouse α-EEA1 | BD Biosciences | 610456 | 1:500 |
| mouse α-p62 | Abcam | Ab56416 | 1:200 |
| rabbit, α-CHMP2B | Abcam | ab33174 | 1:1000 |

**Supplementary Table 6. Secondary antibodies**

| **Antibody** | **Vendor** | **Catalog number** | **Dilution** |
| --- | --- | --- | --- |
| AF568 donkey anti-rabbit | ThermoFisher Scientific | A-10042 | 1:500 |
| AF647 donkey anti-guinea pig | Jackson Immunoresearch | 706-605-148 | 1:500 |
| AF568 doneky anti-mouse | ThermoFisher Scientific | A-10037 | 1:500 |
| AF488 donkey anti-chicken | Jackson Immunoresearch | 703-545-155 | 1:500 |
| AF647 donkey anti-rabbit | ThermoFisher Scientific | A-31573 | 1:500 |

**Supplementary figures**

**
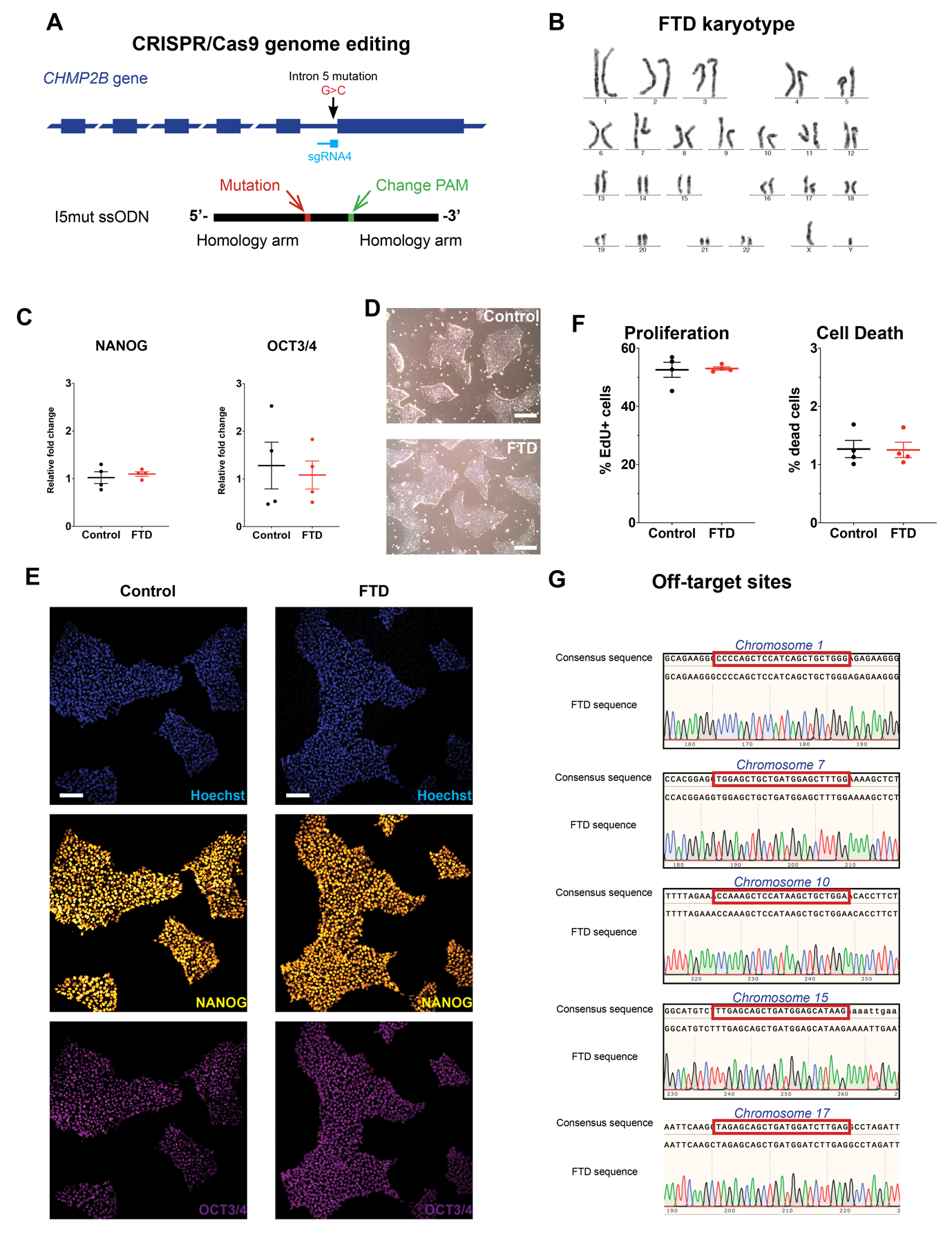
**

**Supplementary Fig. 1. Validation of the CRISPR/Cas9 edited hESC line carrying the intron 5 mutation in the *CHMP2B* gene.** (**A**) Schematic representation of the initial CRISPR/Cas9 strategy to target and modify the CHMP2B gene at the intron 5 (**B**) Results of the karyotype analysis of the FTD-hESC line (**C**) Expression of the pluripotency markers NANOG and OCT3/4 in the FTD-hESC line compared to the Control-hESC line. Data are presented as the mean ± SEM from 4 independent experiments. A two-tailed t-test with Welch’s correction was used to analyze significance (**D**) Representative brightfield images showing typical stem cell colony morphology (**E**) Representative images showing nuclear (Hoechst) localization of NANOG and OCT3/4 protein in both hESC lines, Control (left images) and FTD (right images). (**F**) Results of the proliferation assay showing the percentage of cells labelled with EdU in Control- and FTD-hESC lines (left panel) and results of the cell death assay showing the percentage of dead cells in Control- and FTD-hESC cultures. Data are presented as the mean ± SEM from 4 independent experiments. A two-tailed t-test with Welch’s correction was used to analyze significance (**G**) Sanger sequencing chromatogram of the top-5 predicted off-target sites for the sgRNA4 in the FTD-hESC line. Scale bar = 200 µm (D) 100 µm (E).


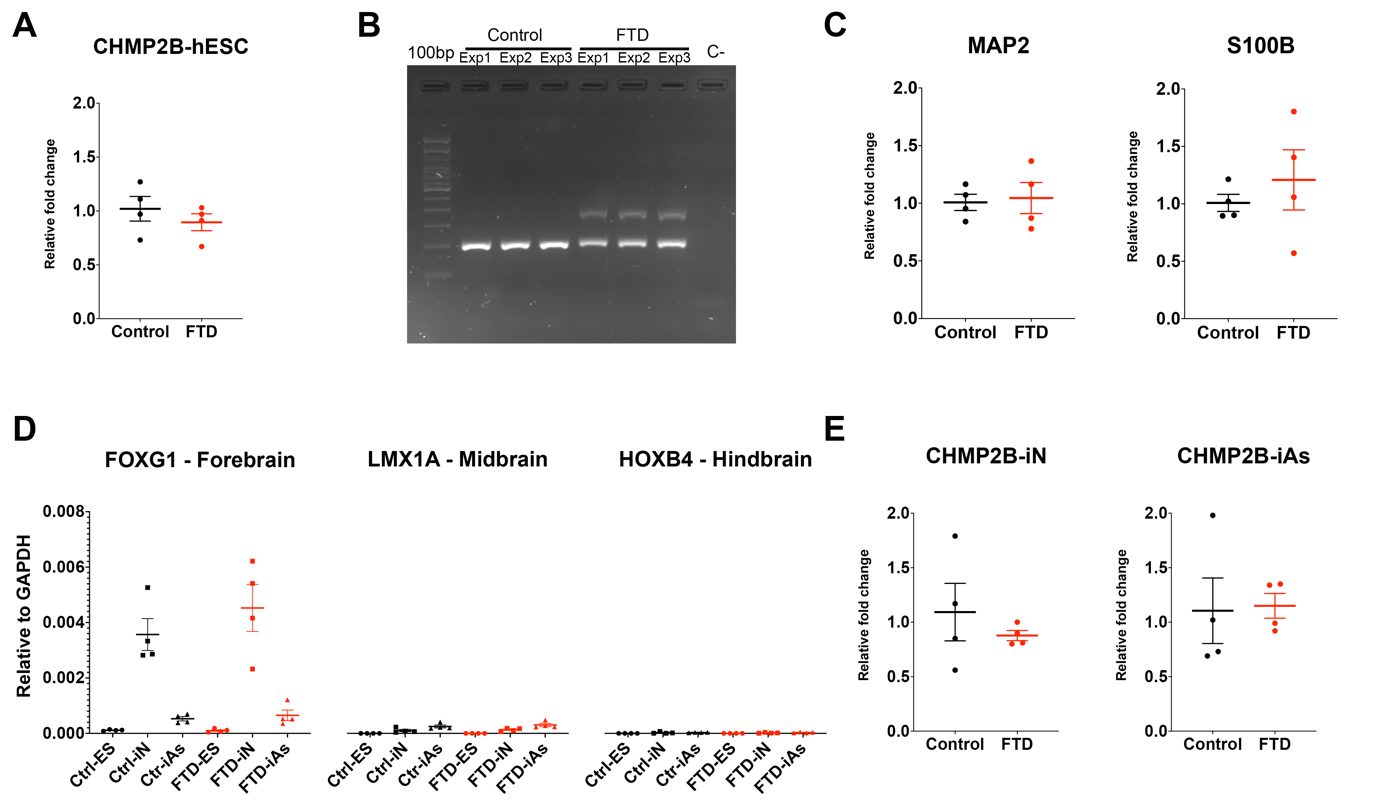


**Supplementary Fig. 2. CHMP2B expression and differentiation towards neurons and astrocytes are not affected by the intron 5 mutation.** (**A**) Expression of CHMP2B in the FTD-hESC line compared to that of the control line. Data are presented as the mean ± SEM from 4 independent experiments. A two-tailed t-test with Welch’s correction was used to analyze significance (**B**) Gel electrophoresis for the PCR amplification of the exon 4 to exon 5 region of the *CHMP2B* mRNA in the Control and FTD-hESC lines. Lower band corresponds to the normal splicing while upper band in FTD lines correspond to the inclusion of intron 5 due to the presence of the mutation. C- = negative control for the PCR. 100bp = ladder. Exp = experiment. Data includes 3 independent experiments. (**C**) Expression of the neuronal marker MAP2 in FTD-iN and the astrocytic markerS100B in FTD-iAs compared to that of the Control-iN and Control-iAs respectively. Data are presented as the mean ± SEM from 4 independent experiments. A two-tailed t-test with Welch’s correction was used to analyze significance (**D**) Expression of the forebrain (FOXG1), midbrain (LMX1A) and hindbrain (HOXB4) markers in iN and iAs derived from the Control and FTD-hESC lines after 7 days of differentiation. Data are presented as the mean ± SEM from 4 independent experiments. (**E**) Expression of CHMP2B in FTD-iN and FTD-iAs compared to that of the Control-iN and Control-iAs respectively. Data are presented as the mean ± SEM from 4 independent experiments. A two-tailed t-test with Welch’s correction was used to analyze significance.


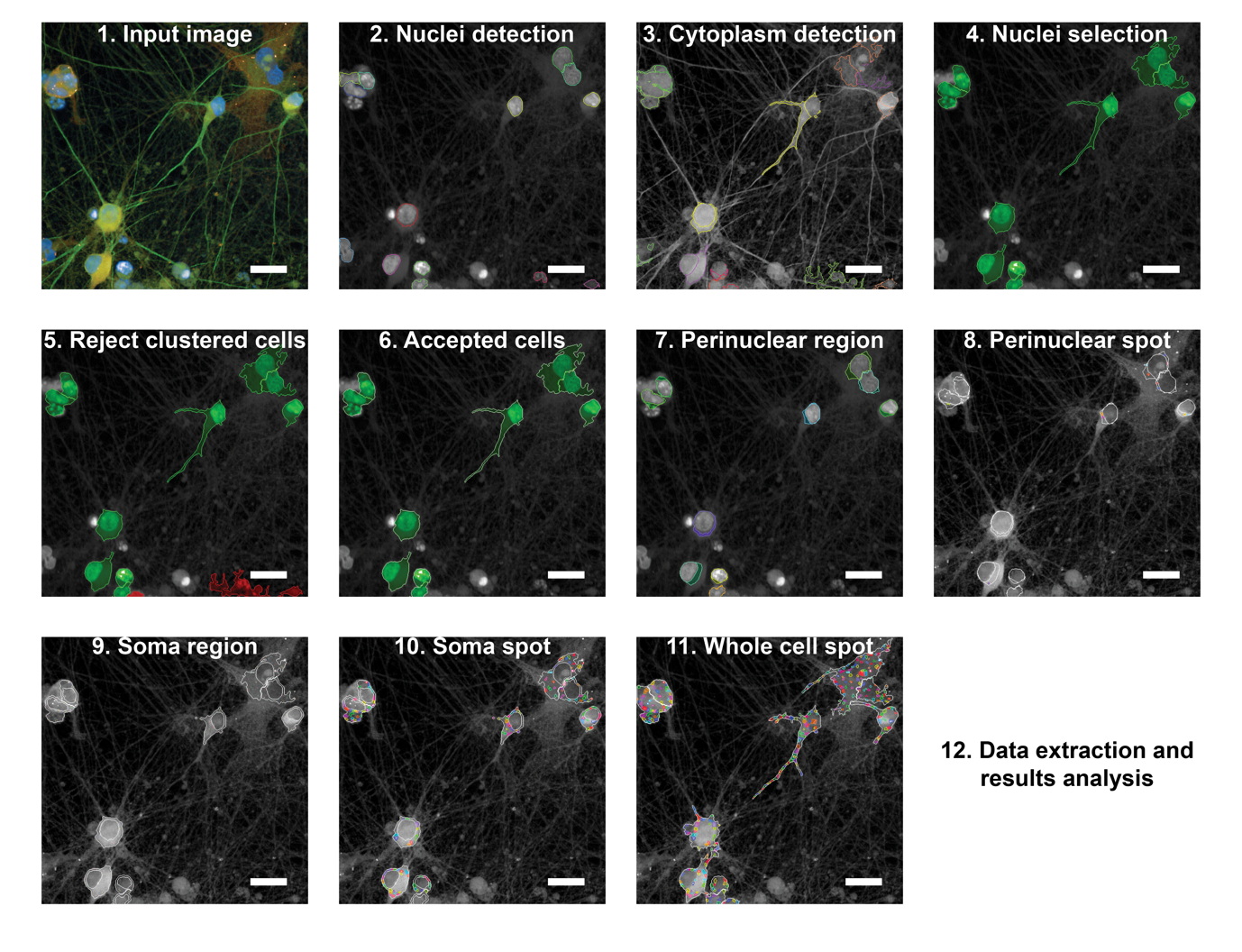


**Supplementary Fig. 3. Workflow of the automatic quantification with the high-content analysis system.** Initial image (1) is used to first automatically detect nuclei based on Hoechst expression (2) and detect cytoplasm based on MAP2 (iN) or GFP (iAs) expression in separate preparations (3). Posteriorly, only nuclei surrounded by cytoplasm are accepted (4) and clustered cells are rejected (5). From accepted cells (6), perinuclear region is detected (7) and number of spots counted and analyzed (8), followed by detection of the soma region (9) and analysis of spots (10), to finalize with analysis of the spots in the whole cell (11) and data extraction (12). Scale bar = 50 µm.

**
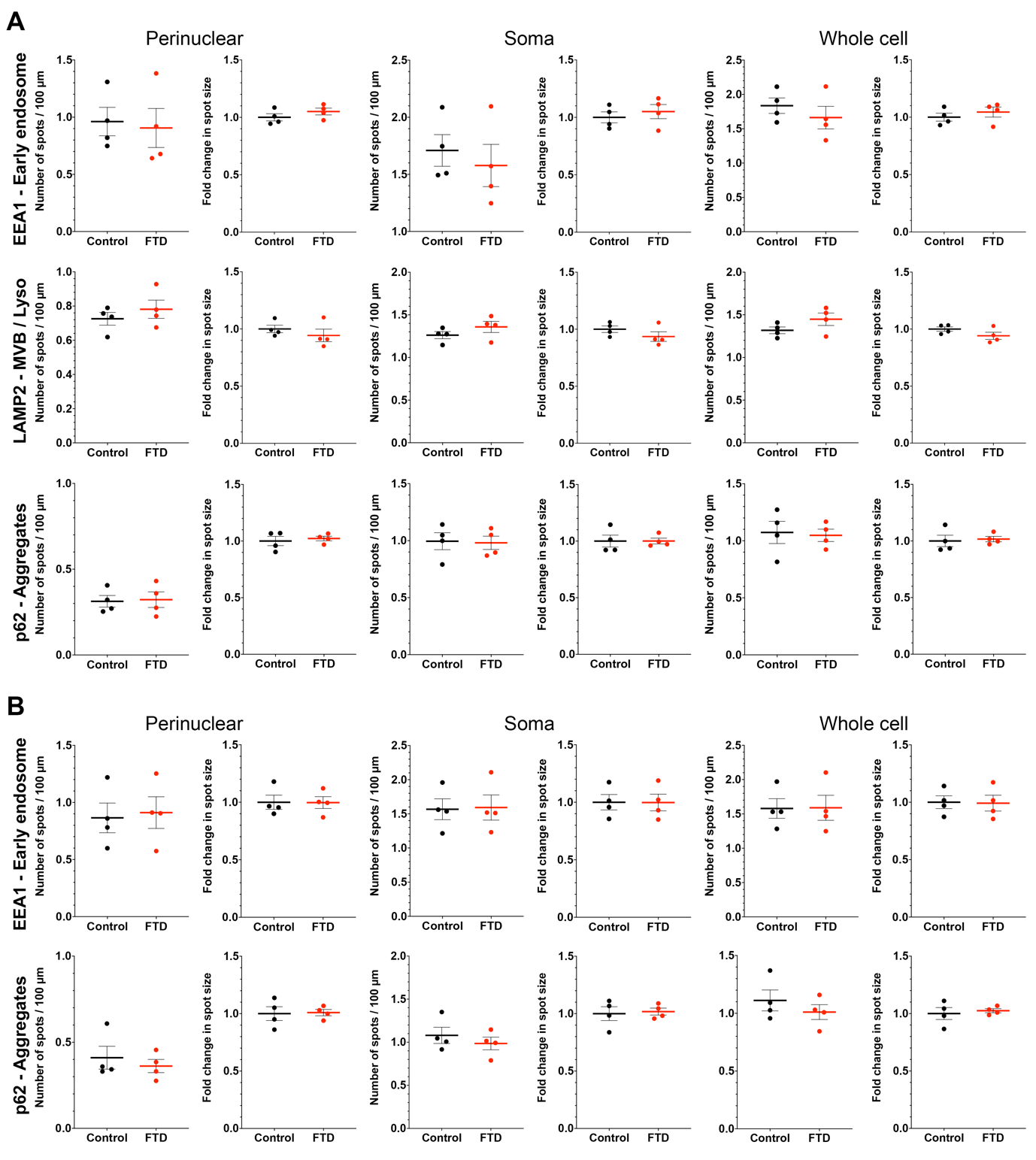
**

**Supplementary Fig. 4. Analysis of the endolysosomal system in co-cultures of FTD neurons and astrocytes.**

(**A, B**) Comparison of the number of dots and change in dot size in the perinuclear and somatic areas as well as in the whole cell of iN (A) and iAs (B) generated from the Control and FTD-hESC lines. EEA1 is used as a marker for early endosomes, LAMP2 as a marker for late endosomes or multivesicular bodies (MVB) and lysosomes (Lyso) and p62 is used as a marker of aggregates and autophagosomes. Data are presented as mean ± SEM from four independent experiments. A two-tailed t-test with Welch’s correction was used to analyze significance and no significant differences were detected.

**
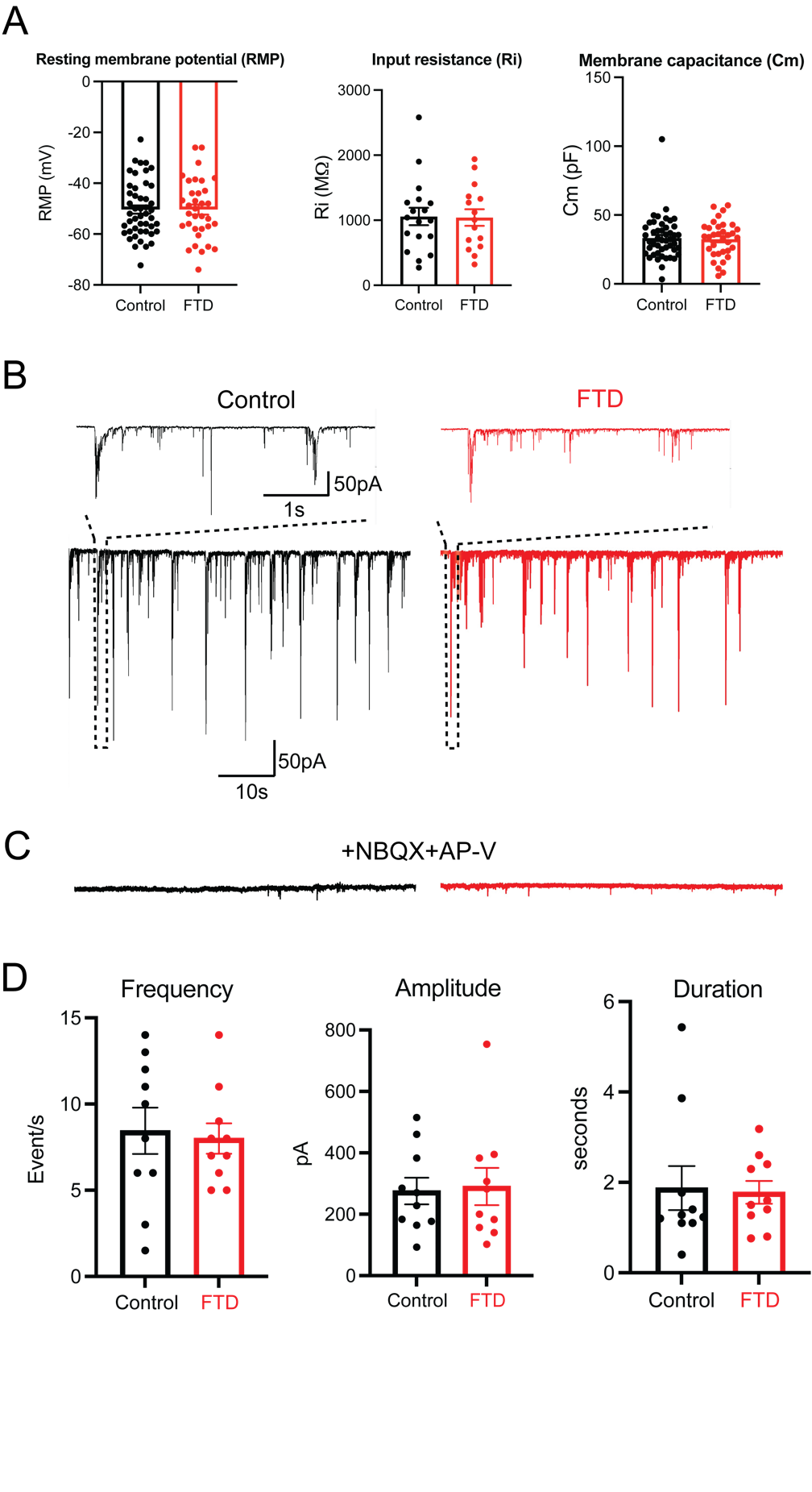
**

**Supplementary Fig. 5. Derived iNs are predominantly excitatory neurons typically with bursting synaptic activity.** **(A)** Resting Membrane Potential (RMP) input resistance (Ri) and capacitance (Cm) values comparing the two iN groups. Data are presented as mean ± SEM of the indicated number of cells from three independent experiments. (**B**) Typical voltage clamp traces of postsynaptic events with magnified insets showing the bursting activity of the two iN cell lines **(C)** Voltage clamp trace recorded from iNs in the presence of NBQX and AP-V, glutamatergic transmission blockers. **(D)** Frequency, amplitude, and duration of the postsynaptic events within recorded bursts in voltage clamp mode. Data are presented as the mean ± SEM. A two-tailed Mann-Whitney test was used to analyze significance.


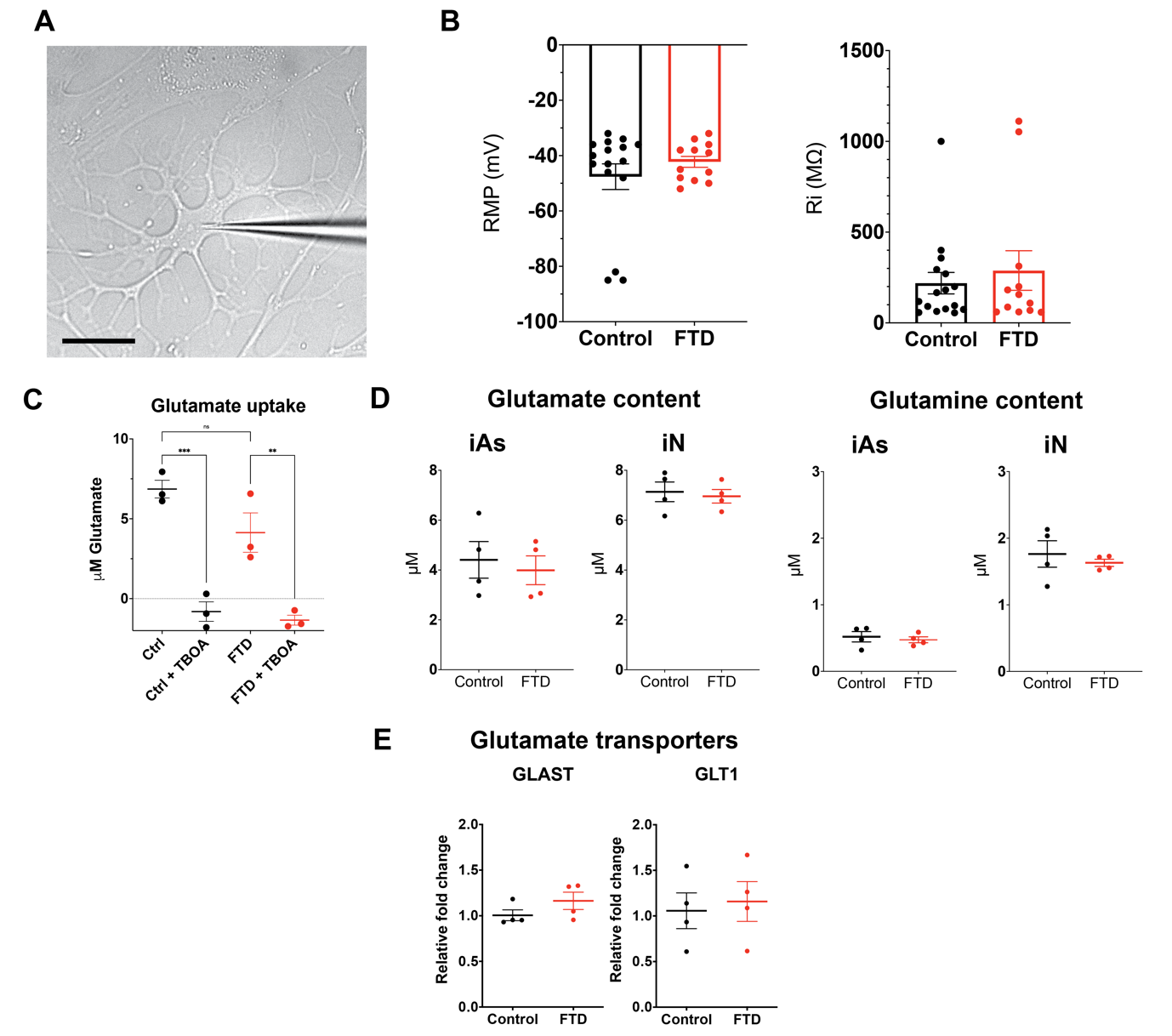


**Supplementary Fig. 6. Glutamate and glutamine content is not affected in FTD-iN and iAs and expression level of glutamate transporters in FTD-iAs is similar to Control-iAs. (A)** Image of a recorded iAs with a patch pipette in monoculture. (**B**) Resting Membrane Potential (RMP) and input resistance (Ri) values comparing the two iAs groups. Data are presented as mean ± SEM of the indicated number of cells from three independent experiments. (**C**) Graph showing results of glutamate uptake assay comparing Control- and FTD-iAs. Data are presented as mean ± SEM from three independent experiments. A one-way ANOVA with multiple comparisons was used to analyze significance. *p ≤ 0.05, **p ≤ 0.01 , ***p ≤ 0.001 (**D**) Results of the concentration of glutamate and glutamine within Control- and FTD-iN and iAs co-cultured with inserts. Data are presented as the mean ± SEM from 4 independent experiments. A two-tailed t-test with Welch’s correction was used to analyze significance (**E**) Results of the expression of the glutamate transporters GLAST and GLT1 in FTD-iAs compared to Control-iAs. Data are presented as the mean ± SEM from 4 independent experiments. A two-tailed t-test with Welch’s correction was used to analyze significance. Scale bar = 50 µm (A).


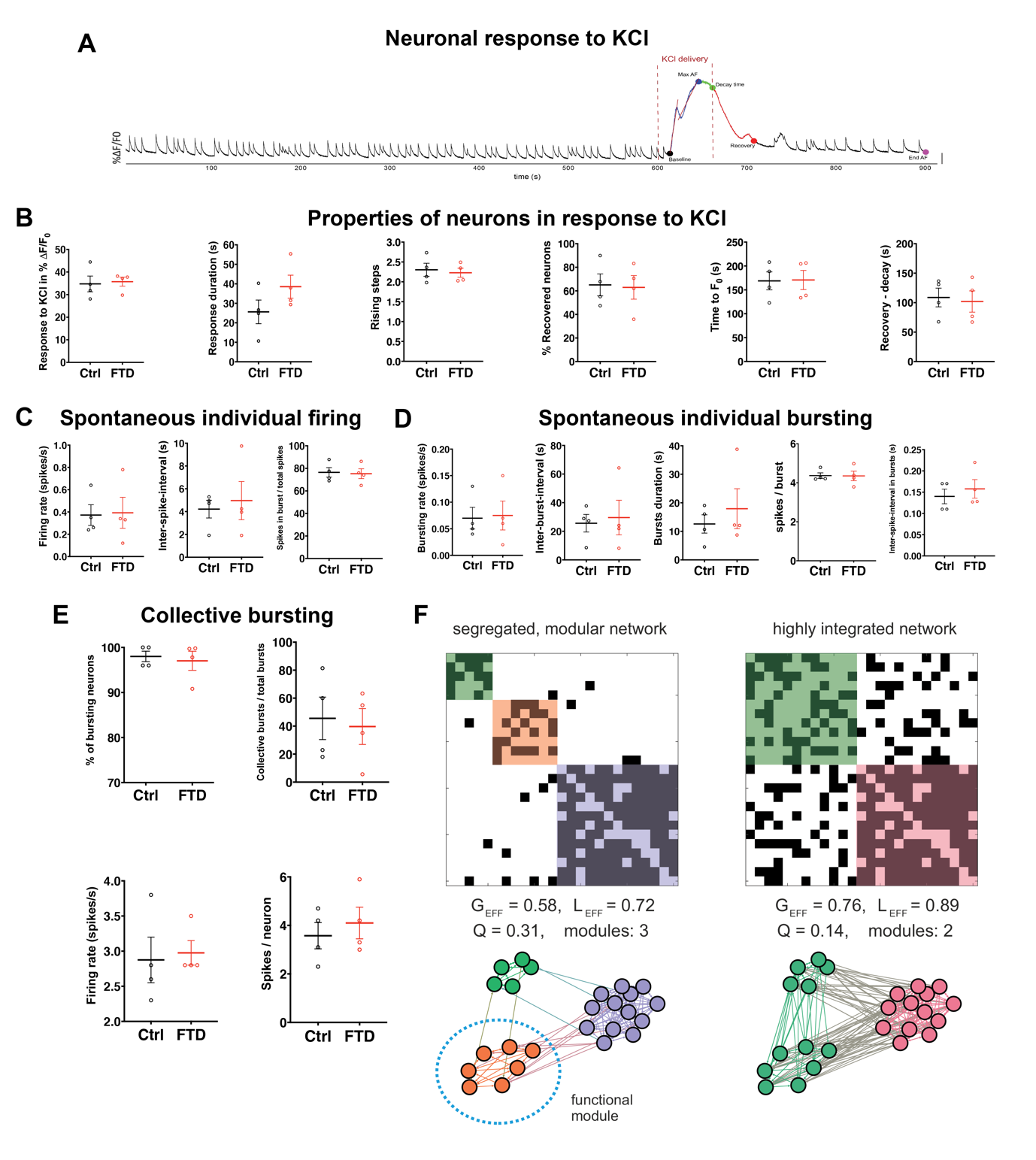


**Supplementary Fig. 7. Calcium imaging analysis of FTD co-cultures did not reveal major differences in individual neuronal activity, response to KCl stimulation, and most collective activity descriptors**. (**A**) Representative calcium imaging recording of a neuron with the KCl stimulation paradigm to examine response to chemical stimulation (**B**) Graphs showing different descriptors of individual neurons after KCl stimulation. These descriptors are the change in fluorescence after stimulation, duration of the response to the stimulation, number of rising steps after stimulation, percentage of neurons achieving pre-stimulation fluorescence levels, time to achieve the pre-stimulation fluorescence levels and the recovery minus the decay time that indicates calcium buffering capacity. Data are presented as the mean ± SEM from 4 independent experiments. A two-tailed t-test with Welch’s correction was used to analyze significance (**C**) Graphs showing spontaneous individual neuron firing properties. These properties are the firing rate indicated by the number of spikes per second, the inter-spike-interval indicating the time in between spikes in seconds and the percentage of total spikes that are part of bursts individual neuronal bursts. Data are presented as the mean ± SEM from 4 independent experiments. A two-tailed t-test with Welch’s correction was used to analyze significance (**D**) Graphs showing properties of spontaneous individual neuron bursts. These properties are the number of bursts per second, the inter-burst-interval indicating the time in between bursts in seconds, the duration in seconds of the bursts, the number of individual spikes within each burst, and the inter-spike-interval within the bursts, indicating the time in seconds between spikes within a single burst. Data are presented as the mean ± SEM from 4 independent experiments. A two-tailed t-test with Welch’s correction was used to analyze significance. (**E**) Graphs showing complementary descriptors of the collective bursting activity in Control and FTD networks. These descriptors are the percentage of neurons that participate in collective bursts, the percentage of total bursts that are considered collective (more than 90% of the neurons participating), the number of spikes per second within bursts in neurons participating in the collective bursts, and the number of spikes per neuron within a collective burst. Data are presented as the mean ± SEM from 4 independent experiments. A two-tailed t-test with Welch’s correction was used to analyze significance. (**F**) Toy networks constituted by 25 neurons to illustrate the network descriptors depicted in Fig. 6(c)-(d) and comparing a segregated network (left) and a highly integrated one (right). The top panels show the connectivity matrices and the bottom ones the corresponding network maps. Each small black square in the matrices is a functional connection and the colored boxes are functional modules. By comparing the matrices and maps, one can observed that, for the segregated network, the number of connections within modules is much higher than across modules, while for the integrated network the number of within and between connections is similar. The network descriptor G_EFF_, L_EFF_ and Q quantify the differences between networks. G_EFF_ and L_EFF_ are associated to global and local average neuronal communication, and are low for the segregated network. Q is associated to the prominence and isolation of the modules and is high for the segregated network. Ctrl = Control.
